# Silylamido supported dinitrogen heterobimetallic complexes: syntheses and their catalytic ability

**DOI:** 10.1093/nsr/nwaa290

**Published:** 2020-12-03

**Authors:** Dan-dan Zhai, Si-jun Xie, Yi Xia, Hua-yi Fang, Zhang-jie Shi

**Affiliations:** Department of Chemistry, Fudan University, Shanghai 200433, China; Department of Chemistry, Fudan University, Shanghai 200433, China; Department of Chemistry, Fudan University, Shanghai 200433, China; Department of Chemistry, Fudan University, Shanghai 200433, China; Department of Chemistry, Fudan University, Shanghai 200433, China; State Key Laboratory of Organometallic Chemistry, Shanghai Institute of Organic Chemistry, Chinese Academy of Sciences, Shanghai 200032, China

**Keywords:** dinitrogen fixation, dinitrogen-metal complex, catalytic ability, disproportionation, isomerization

## Abstract

Molybdenum dinitrogen complexes supported by monodentate arylsilylamido ligand, [Ar(Me_3_Si)N]_3_MoN_2_Mg(THF)_2_[N(SiMe_3_)Ar] (**5**) and [Ar(Me_3_Si)N]_3_MoN_2_SiMe_3_ (**6**) (Ar = 3,5-Me_2_C_6_H_3_) were synthesized and structurally characterized, and proved to be effective catalysts for the disproportionation of cyclohexadienes and isomerization of terminal alkenes. The ^1^H NMR spectrum suggested that the bridging nitrogen ligand remains intact during the catalytic reaction, indicating possible catalytic ability of the Mo-N=N motif.

## Teaser

Dinitrogen heterobimetallic complexes were found to be effective catalysts for the disproportionation of cyclohexadiene and the isomerization of terminal alkenes, in which Mo-N=N motif was considered as a possible catalytic site.

## INTRODUCTION

**Scheme 1.**
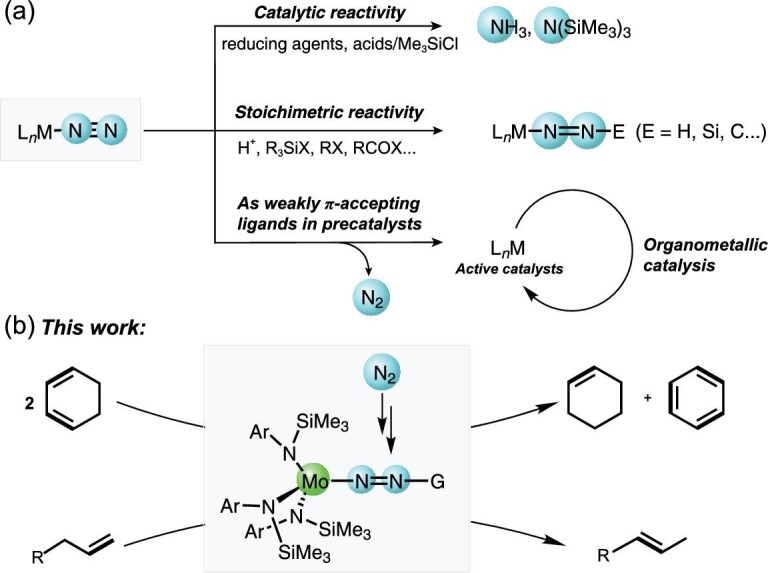
(a) Typical reactivity modes of -N_2_ units in transition metal-dinitrogen complexes. (b) This work: catalytic reactivity of Mo-N_2_ units in the disproportionation of cyclohexadienes and isomerization of terminal alkenes.

Exploring the reactivity of N_2_ units of transition metal-nitrogen complexes is of great significance, but challenging in nitrogen transformation chemistry [1,2]. Since the first Ru-N_2_ complex was prepared in 1965 [3], numerous well-defined transition metal-dinitrogen complexes have been prepared with different bonding modes, showing the potential to activate the inert dinitrogen molecule through coordination chemistry and allowing direct transformation of N_2_ units [4–12]. In many cases, terminal end-on M-N_2_ complexes as the most prevalent bonding mode were proved effective to catalyze reductive reactions of N_2_ to afford ammonia or silylamines [13–16]; moreover, because of the nucleophilicity of the dinitrogen moiety, activated dinitrogen ligands in M-N_2_ (M = Mo [17–28], W [17–19,21,24–26], Fe [29,30], Co [29]) complexes were transformed into N-containing organic compounds with carbon-based electrophiles. On the other hand, late-transition metal-N_2_ complexes (M = Co [31–36], Ru [37–42], Ir [43], Fe [44–48]) have also been reported as precatalysts for organometallic transformations, including cycloaddition and hydrofunctionalization of olefins [31–35,44–48], semihydrogenation of alkynes [36], transfer hydrogenation of ketones [38,39] and acceptorless dehydrogenation of alcohols [40–42]. In these systems, dinitrogen (N_2_) as a weakly π-accepting ligand to stabilize highly reactive and low valence-electron species, was proved not to be involved in the catalytic processes. Actually, there are only a few examples of M-N_2_ units as active sites in catalytic organic transformations. In 2004, Hidai reported that Ti-W heterobimetallic dinitrogen complexes were excellent precursors for copolymerization of ethylene and 1-hexene, in which the W-N_2_ fragment acted as a unique spectator ligand to the catalytically active titanium center [49]. The intriguing results hint at potential reactivity of the coordinated N_2_ units in organometallic catalysis. Herein, we synthesized and structurally characterized molybdenum-nitrogen complexes supported by monodentate arylsilylamido ligand (L = [N(SiMe_3_)Ar]). Meanwhile, we observed catalytic reactivity of the Mo-N_2_ unit as a key motif in disproportionation of cyclohexadienes and isomerization of terminal alkenes where the -N_2_ ligands unusually remain intact. In this catalytic reaction, the Mo-N=N motif was considered as a possible catalytic site to advance the hydrogen transfer (Scheme 1).

Inspired by gradually understanding the structure and mechanism of nitrogenase FeMo–cofactor in the reduction of atmospheric N_2_ [50–54], the chemistry of Mo-N_2_ complexes has been studied for decades, with Mo showing its uniqueness in terms of variable oxidation states and readily forming complexes with N_2_ [55–61]. To investigate the catalytic reactivity of molybdenum complexes with different structure characteristics in N_2_ fixation, various supporting ligands were designed and developed, such as phosphine [17–22,24–26,62,63], triamidoamine [64–68] and pincer [69–72] ligands. Seminal studies by Cummins and co-workers demonstrated the ability of alkylarylamido ligands to stabilize low valent Mo(III) complexes and cleave N_2_ [73,74]. As surrogates of this ligand set, monodentate *N*-aryl-*N*-silylamido ligands have not yet been investigated in a similar system, although -N(SiMe_3_)_2_ [75–78] or silylated multidentate amido ligands -[(R_3_SiNCH_2_CH_2_)_3_N]^3−^ (R_3_Si = Me_3_Si or *^t^*BuMe_2_Si) [79–82], -[N(SiMe_2_CH_2_P*^i^*Pr_2_)_2_]^−^ [83], -[PhP(CH_2_SiMe_2_NSiMe_2_CH_2_)_2_PPh]^2−^ [84] and -[PhP(CH_2_SiMe_2_NPh)_2_]^2−^ [85] have been used to support transition-metal dinitrogen complexes since 1990. We envisaged that the ready introduction of the bulky silyl group would intrinsically tune the steric and electronic coordination sphere of amido donors, inducing new reactivity patterns of coordinated dinitrogen ligands in the Mo-N_2_ complexes.

## RESULTS AND DISCUSSION

**Scheme 2.**
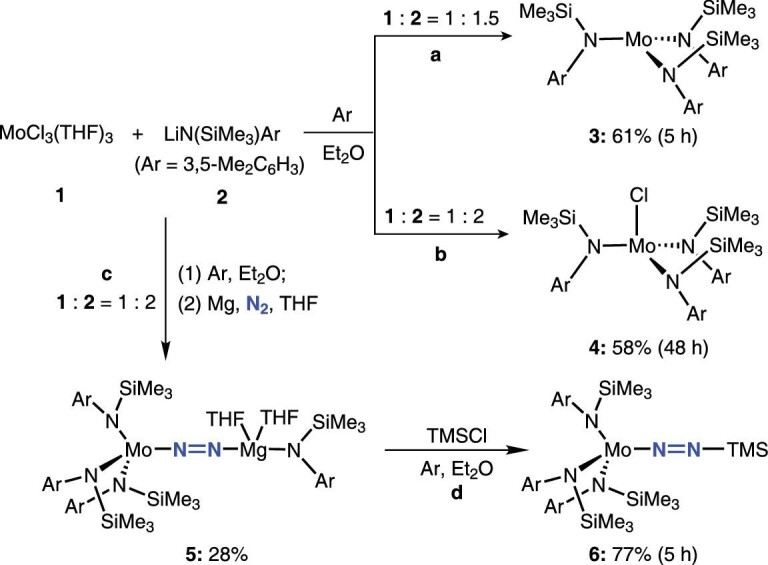
Synthesis of molybdenum complexes **3**–**6**.

**Figure 1.**
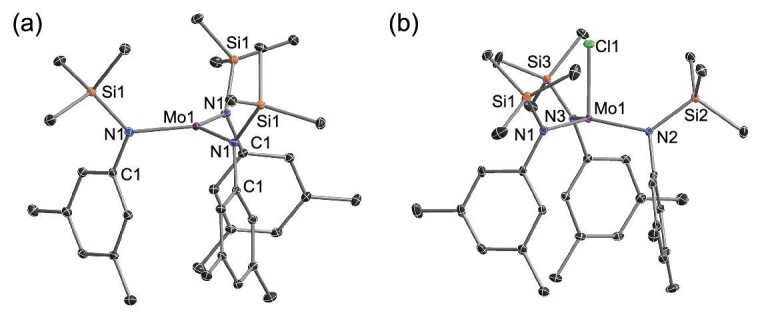
Molecular structures of **3**–**4** with thermal ellipsoids set at 10% probability. (a) Mo[N(SiMe_3_)Ar]_3_, **3**; (b) ClMo[N(SiMe_3_)Ar]_3_, **4**. Hydrogen atoms have been omitted for clarity. Selected bond lengths (Å) and angles (°): **3** Mo1-N1, 1.9854(16); N1-Si1, 1.7507(15); N1-Mo1-N1, 119.755(6); Mo1-N1-Si1, 126.306(112). **4** Mo1-N1, 1.9576(40); Mo1-N2, 1.9635(36); Mo1-N3, 1.9618(38); Mo1-Cl1, 2.3221(16); N1-Mo1-N2, 119.055(160); Cl1-Mo1-N1, 95.977(127).

The reaction of MoCl_3_(THF)_3_ (**1**) with 1.5 equivalents of lithium *N*-(trimethylsilyl)anilide (**2**) in Et_2_O for 5 h afforded the corresponding tris-anilide complex of Mo[N(SiMe_3_)Ar]_3_ (**3**) in moderate yield (Scheme 2, **a**). X-ray diffraction on single crystals revealed that three-coordinate **3** was mononuclear with silylamino substituents arrangement above the trigonal plane of MoN_3_ core (Fig. 1, **a**). Compared with complex Mo[N(*^t^*Bu)Ar]_3_ [73,74], a long Mo-N1 distance (1.985 Å) and small Mo-N-Si bond angles (126°) might arise from the slightly different steric hindrance and electronic pattern of silylamido ligand around the Mo center. We then carried out reaction **3** with N_2_ in the condition for conversion of Mo[N(*^t^*Bu)Ar]_3_ to N≡Mo[N(*^t^*Bu)Ar]_3_ (1 atm of N_2_, *d_8_*-toluene, −35°C). With less electron negativity and the poor electron donating ability of silicon (Si), **3** was proved unreactive with N_2_ molecules even at −35°C for 5 days. Lengthening the reaction time of MoCl_3_(THF)_3_ and lithium amide, the ^1^H NMR spectrum of the crude product mixture showed that Mo^III^**3** had disappeared. The Mo^IV^-Cl complex **4** was observed as the only product, along with a small amount of free ligand HN(SiMe_3_)Ar (Scheme 2, **b**). A similar result was reported by the Fürstner group to isolate complex ClMo[N(*^t^*Bu)Ar]_3_ (Ar = 3,5-dimethoxyphenyl) [86].

**Figure 2.**
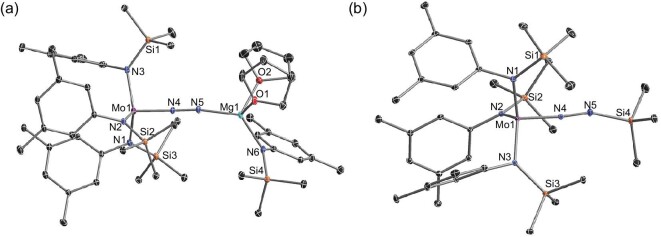
Molecular structures of **5**–**6** with thermal ellipsoids set at 10% probability. (a) [Ar(Me_3_Si) N]_3_MoN_2_Mg(THF)_2_[N(SiMe_3_)Ar], **5**; (b) [Ar(Me_3_Si)N]_3_MoN_2_SiMe_3_, **6**. Hydrogen atoms have been omitted for clarity. Selected bond lengths (Å) and angles (°): **5** Mo1-N1, 1.998(4); Mo1-N2, 2.0066(34); Mo1-N3, 2.0150(39); Mo1-N4, 1.8110(34); N4-N5, 1.1942(48); Mg1-N5, 1.9621(35); Mg1-N6, 1.9924(31); Mo1-N4-N5, 178.107(287); N4-N5-Mg1, 171.026(282). **6** Mo1-N1, 1.9815(14); Mo1-N2, 1.9911(12); Mo1-N3, 1.9785(12); Mo1-N4, 1.7707(12); N4-N5, 1.2139(18); Si4-N5, 1.7092(16); Mo1-N4-N5, 174.306(118); N4-N5-Si4, 160.513(136).

Reduction of the Mo^IV^-Cl reaction mixture with magnesium powder under N_2_ atmosphere (1 atm) produced the Mo-N_2_ complex (Scheme 2, **c**). Diamagnetic signals in the proton NMR spectrum indicated a high oxidation state of the Mo center. X-ray study gave the unambiguous structure of [Ar(Me_3_Si)N]_3_MoN_2_Mg(THF)_2_[N(SiMe_3_)Ar] (**5**), in which Mo and Mg were both supported by silylamido ligand, and bridged by dinitrogen ligand to form the heterobimetallic dinitrogen complex (Fig. 2, **a**). The bond length of N–N is 1.194 Å, indicating the possible feature of N=N double bond, similar to those found in the diazenido species {[N_3_N]Mo-N=N}_2_Mg(THF)_2_ (1.195(13) Å and 1.164(13) Å) [80]. Additionally, **5** could be silylated by trimethylsilyl chloride (Me_3_SiCl) at the β (terminal) nitrogen atom to afford [Ar(Me_3_Si)N]_3_MoN_2_SiMe_3_ (**6**) (Scheme 2, **d**). An X-ray analysis of this complex showed a long N–N bond (1.214 Å) (Fig. 2, **b**). An analogous Mo-N=N-Mg complex supported by other silylamido ligand –N(SiMe_2_^*t*^Bu)Ar was prepared using similar procedures and characterized by X-ray chromatography (Fig. S6). Unfortunately, attempts to obtain analytically pure material failed.

According to those structural features, we envisioned that the hydrazine-like Mo-N=N-Mg backbone (**5**) with synergetic effects of both alkaline-earth metal and transition metal might be a good precursor to transfer two nitrogen atoms to organic molecules through [4 + 2] cycloaddition [87,88]. However, after treatment of **5** with a stoichiometric amount of 1,3-cyclohexadiene (**7**) in C_6_D_6_ at 100°C for 24 h, it was found that featured signals of the complex remained in the ^1^H NMR spectrum of the reaction mixture, implying the remarkable stability of **5** under the reaction conditions. The substrate **7** was completely consumed and two new sets of ^1^H NMR signals appeared, which were a perfect fit for the disproportionation products benzene (**8**) and cyclohexene (**9**) (Fig. S7). Such disproportionation has been studied with different transition-metal catalysts [89–96], but the catalytic ability of complexes with metal-dinitrogen moiety, particularly from N_2_ gas, has not yet been observed. This study could stimulate new avenues to develop efficient catalysts directly from N_2_. On the other hand, identification of the active centers (Mo or Mg) of the bimetallic complex and their exact behaviors also attracted us to unveiling of the catalytic disproportionation.

To prove the catalytic reactivity of **5**, two experimental protocols were followed. Firstly, having confirmed the stoichiometric conversion of 1,3-cyclohexadiene, we added an additional amount of the substrate up to 60 equivalents in portions. Indeed, 90% of **7** converted to **8** and **9** after 18 days (Fig. S8, catalyst/substrate = 0.016/1), showing that the catalyst remained active. We also conducted the reaction by adding 60 equivalents of **7** into the C_6_D_6_ solution of **5** in one portion. After 180 h, the conversion of **7** was 99%, monitored by *in situ* NMR (Fig. S9). A small amount of cyclohexane (**10**) was detected during the process, suggesting potential hydrogen-transfer reduction of non-conjugate alkene with this catalyst. Isomerized 1,4-cyclohexadiene (**11**) was also observed, which could also further transform into **8** and **9** by catalytic disproportionation (Fig. S13). The reaction pathway was different from a previously reported case in which a cationic molybdenum nitride species transferred a hydrogen atom from **11** to afford molybdenum imide complex and **8** in a stoichiometric manner [97].

Attempts were made to gain insight into the catalytic reaction (Table 1). Complex **5** exhibited comparable activity towards the disproportionation under 100°C for 24 h on a 0.30 mmol scale (entry 1), while **6** displayed high catalytic reactivity with 94% conversion within 12 h (entry 2). It was noteworthy that those catalysts remained intact after complete conversion of **7** (Figs S10 and S14), suggesting that the activated -N_2_ units were retained during catalysis. These observations were different from previously reported catalytic transformations in which the electrically neutral and weakly activated N_2_ units were substitutable ligands for substrate binding [31–48]. When performed under an Ar atmosphere or 10 atm of N_2_, reactions also smoothly occurred (entry 3 and 4). Unlike Mo-N_2_ complexes, featured signals of **3** could not be detected after heating at 100°C for a short time (< 5 h) despite its low catalytic competence (entry 5). Related [N]_3_Mo^IV^-Cl complex failed to promote such disproportionation efficiently (entry 6). Magnesium and lithium *N*-(trimethylsilyl)-3,5-dimethylanilides were also tested, but failed (entry 7 and 8). Therefore, these results indicate that Mo-N=N moiety was a key structure and the active site was located at the Mo center. Kinetic studies showed that the initial rate of disproportionation dependent on the concentration of catalyst **5** was first order (Fig. S25), further evidence that **5** was not a precatalyst in the transformation.

**Table 1.** Catalytic disproportionation reaction of 1,3-cyclohexadiene^a^.

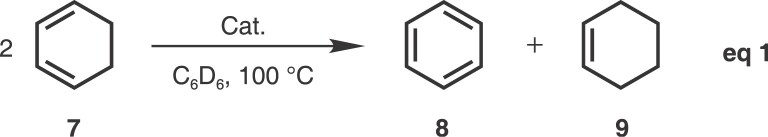		
Entry	Catalysts	Conv. (%)^a,b^
1	**5** (24 h)	99%
2	**6** (12 h)	94%
3	**5** (24 h), Ar	98%^c^
4	**5** (24 h), N_2_ (10 atm)	99%^d^
5	**3** (24 h)	15%
6	**4** (24 h)	13%
7	Mg[N(SiMe_3_)Ar]_2_ (36 h)	NR
8	Li[N(SiMe_3_)Ar] (19 h)	NR

^a^Conditions: 1,3-cyclohexadiene (0.30 mmol), catalyst (10 mol%), 100 °C, C_6_D_6_ (0.5 mL), N_2_ atmosphere (1 atm). ^b^Determined by ^1^H NMR spectroscopy. ^c^In an Ar atmosphere. ^d^The reaction was carried out in an autoclave pressurized with 10 atm of N_2_.

**Scheme 3.**
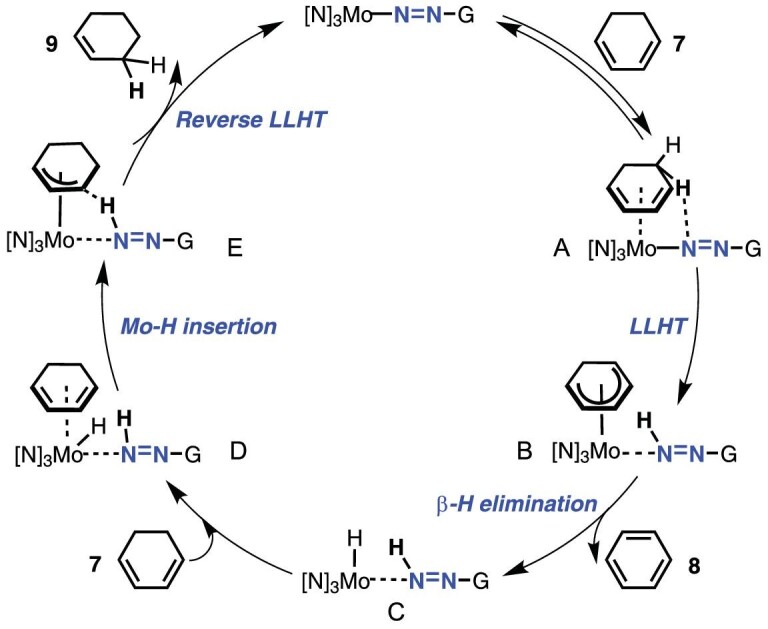
Proposed mechanism for the disproportionation of 1,3-cyclohexadiene.

Based on these observations, we proposed a plausible catalytic pathway shown in Scheme 3. After coordination of diene **7** to the Mo center (**A**), the resulting activated allylic hydrogen was transferred from 1,3-cyclohexadiene to N_α_ atom through ligand-to-ligand hydrogen transfer (LLHT) [98–101], to form a cyclohexadienyl-Mo complex (**B**) with hydrazine as a ligand. β-hydride elimination of **B** released benzene and afforded the key intermediate Mo-H species (**C**), which coordinated with another molecule of 1,3-cyclohexadiene with subsequent insertion (or hydromolybdation) to generate a cyclohexenyl-Mo species (**E**), further undergoing reverse LLHT to produce cyclohexene and regenerate the catalyst.

**Scheme 4.**
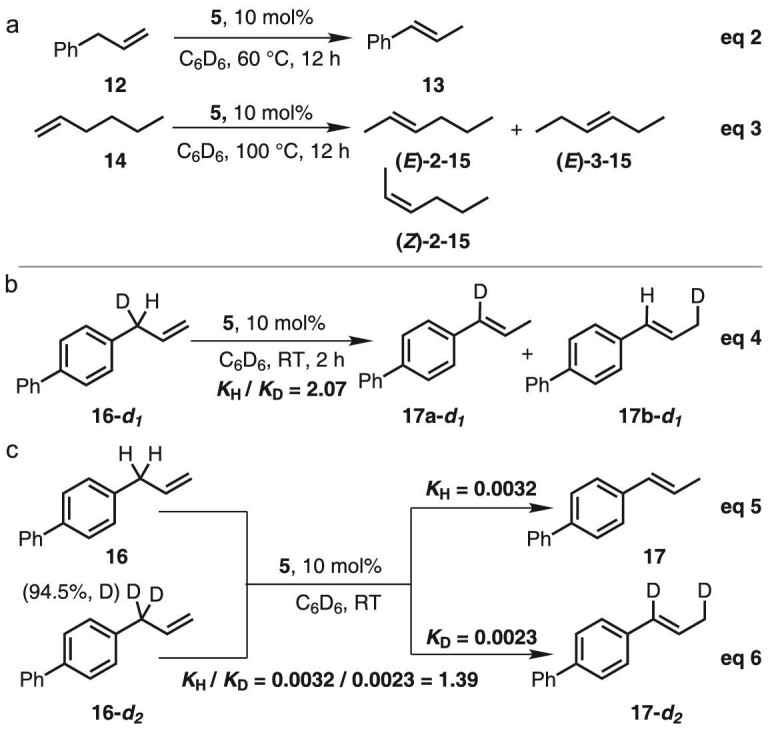
Catalytic isomerization of terminal alkenes and kinetic isotope effect experiment.

According to the above proposal, complex **5** should be suitable to catalyze alkene isomerization through LLHT-reverse LLHT process. We examined allylbenzene (**12**) as substrate and found that the isomerization indeed took place at high efficiency to afford thermodynamic *trans*-adduct **13** as the product (Scheme 4, eq 2). 1-Hexene (**14**) was also submitted to the reaction system and internal alkenes were produced, albeit with poor site- and regio-selectivity (Scheme 4, eq 3). Kinetic studies indicated that C-H cleavage was not involved in the rate-determining step (Scheme 4, eq 4–6), consistent with the feature of hydrogen transfer between ligands [99].

## CONCLUSIONS

In summary, we demonstrated that monodentate silylamido substituents could serve as excellent ligands for supporting low-valence complexes [N]_3_Mo and heterobimetallic dinitrogen complexes [N]_3_Mo-N_2_-Mg[N], which showed great catalytic ability in the disproportionation of cyclohexediene and isomerization of terminal alkenes with -N_2_ ligands intact. Preliminary mechanistic studies indicated that the active catalytic center was the Mo-N_2_ moiety through a ligand-to-ligand hydrogen transfer process. The detailed mechanism and new catalytic applications of these M-N_2_ complexes in organic transformations are currently under consideration.

## DATA AVAILABILITY

The X-ray crystallographic coordinates for the structures of **3**, **4**, **5**, **6** and Mg(THF)_2_[N(SiMe_3_)Ar]_2_ reported in this paper have been deposited at the Cambridge Crystallographic Data Centre (CCDC) under deposition numbers 1963795, 1963701, 1963702, 1963703 and 1963706. These data can be obtained free of charge from http://www.ccdc.cam.ac.uk/data_request/cif.

## Supplementary Material

nwaa290_Supplemental_Files
