# Supplementary material for: Silylamido supported dinitrogen heterobimetallic complexes: syntheses and their catalytic ability
Source: Natl Sci Rev. 2020 Dec 3;8(12):nwaa290. doi: 10.1093/nsr/nwaa290 (PMC8694672; doi:10.1093/nsr/nwaa290)

checkCIF/PLATON report

Structure factors have been supplied for datablock(s) xsj_MoCl_9

THIS REPORT IS FOR GUIDANCE ONLY. IF USED AS PART OF A REVIEW PROCEDURE FOR PUBLICATION, IT SHOULD NOT REPLACE THE EXPERTISE OF AN EXPERIENCED CRYSTALLOGRAPHIC REFEREE.

No syntax errors found. CIF dictionary Interpreting this report

Datablock: xsj_MoCl_9

```
Bond precision:  C-C = 0.0080 A          Wavelength=0.71073

Cell:            a=35.1737(14)     b=35.6535(14)     c=24.6351(11)
                 alpha=90          beta=90           gamma=90
Temperature:     150 K

                 Calculated                   Reported
Volume           30894(2)                     30894(2)
Space group      F d d 2                      F d d 2
Hall group       F 2 -2d                      F 2 -2d
Moiety formula   C33 H54 Cl Mo N3 Si3         C33 H54 Cl Mo N3 Si3
Sum formula      C33 H54 Cl Mo N3 Si3         C33 H54 Cl Mo N3 Si3
Mr               708.45                       708.45
Dx,g cm-3        1.219                        1.219
Z                32                           32
Mu (mm-1)        0.526                        0.526
F000             11968.0                      11968.0
F000’            11927.79
h,k,lmax         46,47,32                     46,47,32
Nref             19248[ 9843]                 19184
Tmin,Tmax        0.881,0.910                  0.850,0.910
Tmin’            0.881

Correction method= # Reported T Limits: Tmin=0.850 Tmax=0.910
AbsCorr = MULTI-SCAN

Data completeness= 1.95/1.00      Theta(max)= 28.320

R(reflections)= 0.0357( 17324)    wR2(reflections)= 0.0844( 19184)

S = 1.138                  Npar= 960
```

The following ALERTS were generated. Each ALERT has the format
test-name_ALERT_alert-type_alert-level.
Click on the hyperlinks for more details of the test.

Alert level C

```
PLAT242_ALERT_2_C Low    'MainMol' Ueq as Compared to Neighbors of        Si1 Check
PLAT242_ALERT_2_C Low    'MainMol' Ueq as Compared to Neighbors of        Si3 Check
PLAT910_ALERT_3_C Missing # of FCF Reflection(s) Below Theta(Min).         10 Note
```

Alert level G

```
PLAT002_ALERT_2_G Number of Distance or Angle Restraints on AtSite         78 Note
PLAT083_ALERT_2_G SHELXL Second Parameter in WGHT  Unusually Large     145.24 Why ?
PLAT171_ALERT_4_G The CIF-Embedded .res File Contains EADP Records          8 Report
PLAT175_ALERT_4_G The CIF-Embedded .res File Contains SAME Records          6 Report
PLAT176_ALERT_4_G The CIF-Embedded .res File Contains SADI Records          6 Report
PLAT301_ALERT_3_G Main Residue  Disorder ..............(Resd  2  )       100% Note
PLAT302_ALERT_4_G Anion/Solvent/Minor-Residue Disorder (Resd  3  )       100% Note
PLAT304_ALERT_4_G Non-Integer Number of Atoms in ...... Resd  2         84.38 Check
PLAT304_ALERT_4_G Non-Integer Number of Atoms in ...... Resd  3         10.62 Check
PLAT380_ALERT_4_G Incorrectly? Oriented X(sp2)-Methyl Moiety .....        C29 Check
PLAT413_ALERT_2_G Short Inter XH3 .. XHn     H8C      ..H66F     .       2.10 Ang.
                                                      x,y,z  =      1_555 Check
PLAT413_ALERT_2_G Short Inter XH3 .. XHn     H10C     ..H66D     .       2.09 Ang.
                                                      x,y,z  =      1_555 Check
PLAT413_ALERT_2_G Short Inter XH3 .. XHn     H20C     ..H55F     .       2.05 Ang.
                                             -1/2+x,y,1/2+z  =      9_455 Check
PLAT413_ALERT_2_G Short Inter XH3 .. XHn     H30C     ..H55E     .       2.08 Ang.
                                         -1/4+x,5/4-y,3/4+z  =     11_465 Check
PLAT722_ALERT_1_G Angle   Calc      108.00, Rep      109.50 Dev...       1.50 Degree
              H40D    -C40B    -H40E       1.555  1.555  1.555     #  462 Check
PLAT794_ALERT_5_G Tentative Bond Valency for Mo1       (IV)      .       4.40 Info
PLAT811_ALERT_5_G No ADDSYM Analysis: Too Many Excluded Atoms ....          ! Info
PLAT860_ALERT_3_G Number of Least-Squares Restraints .............         95 Note
PLAT912_ALERT_4_G Missing # of FCF Reflections Above STh/L=  0.600         22 Note
PLAT913_ALERT_3_G Missing # of Very Strong Reflections in FCF ....          2 Note
PLAT933_ALERT_2_G Number of OMIT Records in Embedded .res File ...          1 Note
PLAT978_ALERT_2_G Number C-C Bonds with Positive Residual Density.          1 Info
```

---

0 **ALERT level A** = Most likely a serious problem - resolve or explain
0 **ALERT level B** = A potentially serious problem, consider carefully
3 **ALERT level C** = Check. Ensure it is not caused by an omission or oversight
22 **ALERT level G** = General information/check it is not something unexpected

1 ALERT type 1 CIF construction/syntax error, inconsistent or missing data
10 ALERT type 2 Indicator that the structure model may be wrong or deficient
4 ALERT type 3 Indicator that the structure quality may be low
8 ALERT type 4 Improvement, methodology, query or suggestion
2 ALERT type 5 Informative message, check

It is advisable to attempt to resolve as many as possible of the alerts in all categories. Often the minor alerts point to easily fixed oversights, errors and omissions in your CIF or refinement strategy, so attention to these fine details can be worthwhile. In order to resolve some of the more serious problems it may be necessary to carry out additional measurements or structure refinements. However, the purpose of your study may justify the reported deviations and the more serious of these should normally be commented upon in the discussion or experimental section of a paper or in the "special_details" fields of the CIF. checkCIF was carefully designed to identify outliers and unusual parameters, but every test has its limitations and alerts that are not important in a particular case may appear. Conversely, the absence of alerts does not guarantee there are no aspects of the results needing attention. It is up to the individual to critically assess their own results and, if necessary, seek expert advice.

Publication of your CIF in IUCr journals

A basic structural check has been run on your CIF. These basic checks will be run on all CIFs submitted for publication in IUCr journals (*Acta Crystallographica*, *Journal of Applied Crystallography*, *Journal of Synchrotron Radiation*); however, if you intend to submit to *Acta Crystallographica Section C* or *E* or *IUCrData*, you should make sure that full publication checks are run on the final version of your CIF prior to submission.

Publication of your CIF in other journals

Please refer to the *Notes for Authors* of the relevant journal for any special instructions relating to CIF submission.

PLATON version of 07/08/2019; check.def file version of 30/07/2019

Datablock xsj_MoCl_9 - ellipsoid plot

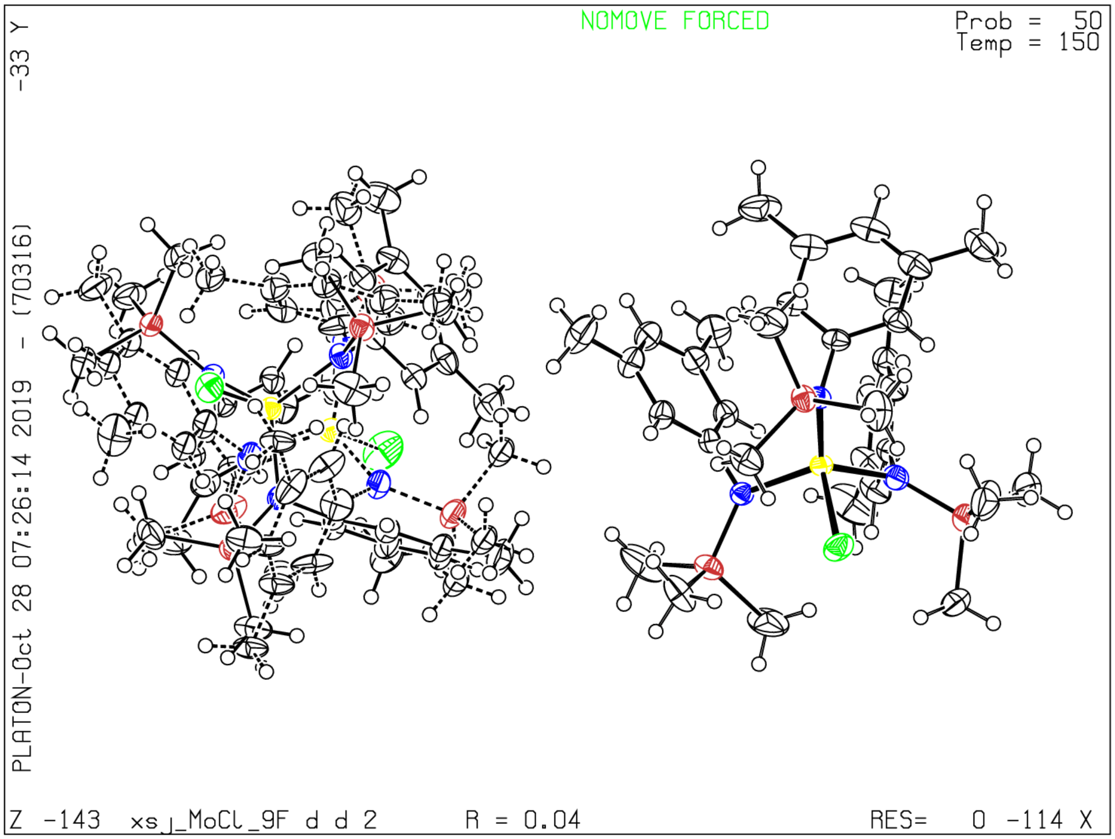

Supplement: nwaa290_Supplemental_Files [file nwaa290_supplemental_files.zip › Complex 4.pdf]
