# Supplementary material for: Silylamido supported dinitrogen heterobimetallic complexes: syntheses and their catalytic ability
Source: Natl Sci Rev. 2020 Dec 3;8(12):nwaa290. doi: 10.1093/nsr/nwaa290 (PMC8694672; doi:10.1093/nsr/nwaa290)

checkCIF/PLATON report

Structure factors have been supplied for datablock(s) 20190214

THIS REPORT IS FOR GUIDANCE ONLY. IF USED AS PART OF A REVIEW PROCEDURE FOR PUBLICATION, IT SHOULD NOT REPLACE THE EXPERTISE OF AN EXPERIENCED CRYSTALLOGRAPHIC REFEREE.

No syntax errors found. CIF dictionary Interpreting this report

Datablock: 20190214

```
Bond precision:  C-C = 0.0075 A          Wavelength=1.54178

Cell:           a=11.2390(4)      b=15.8850(5)     c=17.6581(6)
                alpha=82.436(2)   beta=84.706(2)   gamma=72.222(2)
Temperature:    150 K

                Calculated                 Reported
Volume          2971.33(18)                2971.33(18)
Space group     P -1                       P -1
Hall group      -P 1                       -P 1
Moiety formula  C52 H88 Mg Mo N6 O2 Si4    C52 H88 Mg Mo N6 O2 Si4
Sum formula     C52 H88 Mg Mo N6 O2 Si4    C52 H88 Mg Mo N6 O2 Si4
Mr              1061.90                    1061.89
Dx,g cm-3       1.187                      1.187
Z               2                          2
Mu (mm-1)       2.993                      2.993
F000            1136.0                     1136.0
F000'           1140.48
h,k,lmax        13,19,21                   13,19,21
Nref            11374                      11239
Tmin,Tmax       0.750,0.942                0.750,0.940
Tmin'           0.741

Correction method= # Reported T Limits: Tmin=0.750 Tmax=0.940
AbsCorr = MULTI-SCAN

Data completeness= 0.988          Theta(max)= 70.430

R(reflections)= 0.0629( 9195)     wR2(reflections)= 0.1934( 11239)

S = 1.108                   Npar= 644
```

The following ALERTS were generated. Each ALERT has the format
test-name_ALERT_alert-type_alert-level.
Click on the hyperlinks for more details of the test.

Alert level C

```
PLAT220_ALERT_2_C Non-Solvent  Resd 1  C   Ueq(max)/Ueq(min) Range        3.2 Ratio
PLAT234_ALERT_4_C Large Hirshfeld Difference C45      --C46B     .       0.20 Ang.
PLAT234_ALERT_4_C Large Hirshfeld Difference C47A     --C48A     .       0.17 Ang.
PLAT241_ALERT_2_C High   'MainMol' Ueq as Compared to Neighbors of        C52 Check
PLAT242_ALERT_2_C Low    'MainMol' Ueq as Compared to Neighbors of        Si1 Check
PLAT242_ALERT_2_C Low    'MainMol' Ueq as Compared to Neighbors of         O1 Check
PLAT242_ALERT_2_C Low    'MainMol' Ueq as Compared to Neighbors of         O2 Check
PLAT360_ALERT_2_C Short  C(sp3)-C(sp3) Bond  C51      - C52      .       1.42 Ang.
PLAT906_ALERT_3_C Large K Value in the Analysis of Variance ......      2.060 Check
PLAT911_ALERT_3_C Missing FCF Refl Between Thmin & STh/L=    0.600         47 Report
PLAT972_ALERT_2_C Check Calcd Resid. Dens.  0.90A   From Mo1            -1.54 eA-3
PLAT977_ALERT_2_C Check Negative Difference Density on H51A             -0.31 eA-3
PLAT977_ALERT_2_C Check Negative Difference Density on H51B             -0.38 eA-3
PLAT978_ALERT_2_C Number C-C Bonds with Positive Residual Density.          0 Info
```

Alert level G

```
PLAT002_ALERT_2_G Number of Distance or Angle Restraints on AtSite          7 Note
PLAT003_ALERT_2_G Number of Uiso or Uij Restrained non-H Atoms ...          3 Report
PLAT066_ALERT_1_G Predicted and Reported Tmin&Tmax Range Identical          ? Check
PLAT072_ALERT_2_G SHELXL First  Parameter in WGHT  Unusually Large       0.13 Report
PLAT154_ALERT_1_G The s.u.'s on the Cell Angles are Equal ..(Note)      0.002 Degree
PLAT175_ALERT_4_G The CIF-Embedded .res File Contains SAME Records          1 Report
PLAT176_ALERT_4_G The CIF-Embedded .res File Contains SADI Records          2 Report
PLAT177_ALERT_4_G The CIF-Embedded .res File Contains DELU Records          1 Report
PLAT178_ALERT_4_G The CIF-Embedded .res File Contains SIMU Records          1 Report
PLAT186_ALERT_4_G The CIF-Embedded .res File Contains ISOR Records          1 Report
PLAT301_ALERT_3_G Main Residue  Disorder ..............(Resd  1  )         5% Note
PLAT720_ALERT_4_G Number of Unusual/Non-Standard Labels ..........          1 Note
PLAT779_ALERT_4_G Suspect or Irrelevant (Bond) Angle(s) in CIF . #         67 Check
              N6   -SI04 -MG1     1.555   1.555   1.555            30.61 Deg.
PLAT794_ALERT_5_G Tentative Bond Valency for Mo1       (V)       .       4.73 Info
PLAT860_ALERT_3_G Number of Least-Squares Restraints .............         29 Note
PLAT883_ALERT_1_G No Info/Value for _atom_sites_solution_primary .     Please Do !
PLAT912_ALERT_4_G Missing # of FCF Reflections Above STh/L=  0.600         88 Note
PLAT992_ALERT_5_G Repd & Actual _reflns_number_gt Values Differ by          2 Check
```

```
   0 ALERT level A = Most likely a serious problem - resolve or explain
   0 ALERT level B = A potentially serious problem, consider carefully
  14 ALERT level C = Check. Ensure it is not caused by an omission or oversight
  18 ALERT level G = General information/check it is not something unexpected

   3 ALERT type 1 CIF construction/syntax error, inconsistent or missing data
  13 ALERT type 2 Indicator that the structure model may be wrong or deficient
   4 ALERT type 3 Indicator that the structure quality may be low
  10 ALERT type 4 Improvement, methodology, query or suggestion
   2 ALERT type 5 Informative message, check
```

It is advisable to attempt to resolve as many as possible of the alerts in all categories. Often the minor alerts point to easily fixed oversights, errors and omissions in your CIF or refinement strategy, so attention to these fine details can be worthwhile. In order to resolve some of the more serious problems it may be necessary to carry out additional measurements or structure refinements. However, the purpose of your study may justify the reported deviations and the more serious of these should normally be commented upon in the discussion or experimental section of a paper or in the "special_details" fields of the CIF. checkCIF was carefully designed to identify outliers and unusual parameters, but every test has its limitations and alerts that are not important in a particular case may appear. Conversely, the absence of alerts does not guarantee there are no aspects of the results needing attention. It is up to the individual to critically assess their own results and, if necessary, seek expert advice.

Publication of your CIF in IUCr journals

A basic structural check has been run on your CIF. These basic checks will be run on all CIFs submitted for publication in IUCr journals (*Acta Crystallographica*, *Journal of Applied Crystallography*, *Journal of Synchrotron Radiation*); however, if you intend to submit to *Acta Crystallographica Section C* or *E* or *IUCrData*, you should make sure that full publication checks are run on the final version of your CIF prior to submission.

Publication of your CIF in other journals

Please refer to the *Notes for Authors* of the relevant journal for any special instructions relating to CIF submission.

PLATON version of 07/08/2019; check.def file version of 30/07/2019

Datablock 20190214 - ellipsoid plot

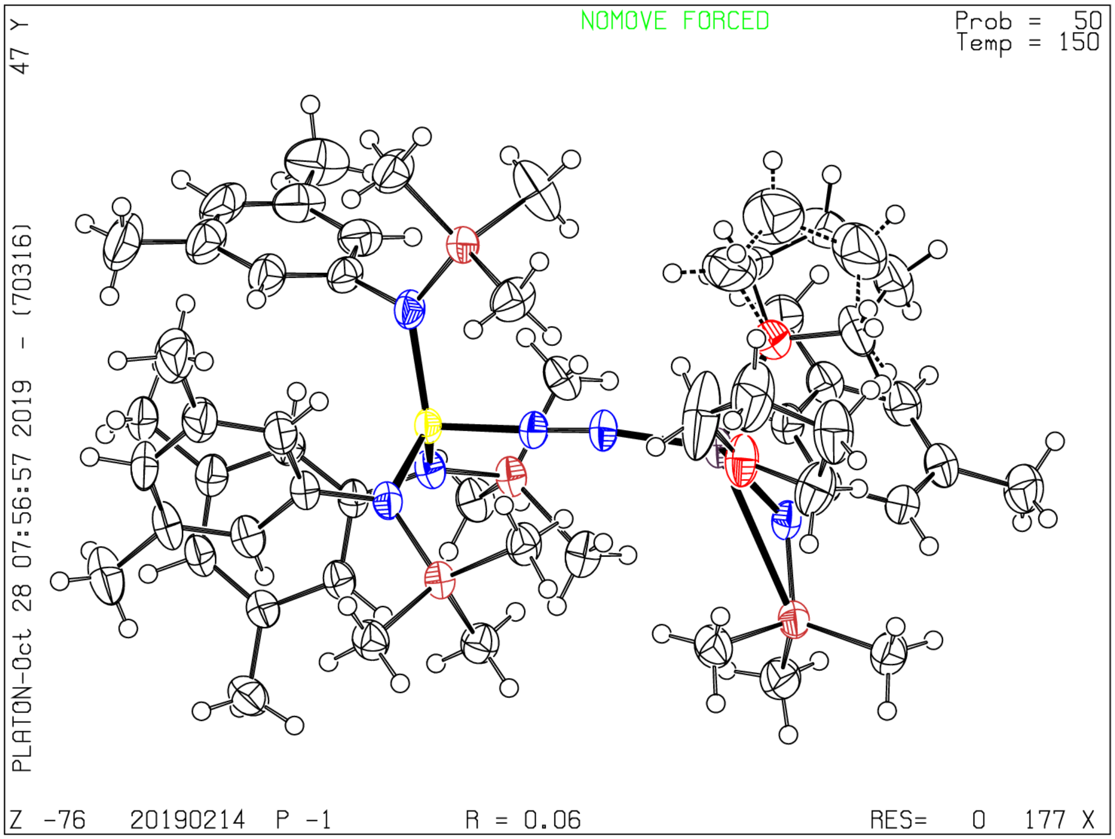

Supplement: nwaa290_Supplemental_Files [file nwaa290_supplemental_files.zip › Complex 5.pdf]
