# Supplementary material for: Silylamido supported dinitrogen heterobimetallic complexes: syntheses and their catalytic ability
Source: Natl Sci Rev. 2020 Dec 3;8(12):nwaa290. doi: 10.1093/nsr/nwaa290 (PMC8694672; doi:10.1093/nsr/nwaa290)

checkCIF/PLATON report

Structure factors have been supplied for datablock(s) ZDD_04_03

THIS REPORT IS FOR GUIDANCE ONLY. IF USED AS PART OF A REVIEW PROCEDURE FOR PUBLICATION, IT SHOULD NOT REPLACE THE EXPERTISE OF AN EXPERIENCED CRYSTALLOGRAPHIC REFEREE.

No syntax errors found. CIF dictionary Interpreting this report

Datablock: ZDD_04_03

```
Bond precision:  C-C = 0.0021 A          Wavelength=0.71073

Cell:            a=10.4893(3)     b=21.2204(6)       c=19.9158(6)
                 alpha=90         beta=99.828(1)     gamma=90
Temperature:     150 K

                Calculated                 Reported
Volume          4368.0(2)                  4367.9(2)
Space group     P 21/n                     P 1 21/n 1
Hall group      -P 2yn                     -P 2yn
Moiety formula  C36 H63 Mo N5 Si4          C36 H63 Mo N5 Si4
Sum formula     C36 H63 Mo N5 Si4          C36 H63 Mo N5 Si4
Mr              774.21                     774.21
Dx,g cm-3       1.177                      1.177
Z               4                          4
Mu (mm-1)       0.439                      0.439
F000            1648.0                     1648.0
F000'           1642.73
h,k,lmax        14,28,26                   13,28,26
Nref            10869                      10839
Tmin,Tmax       0.900,0.924                0.860,0.930
Tmin'           0.900

Correction method= # Reported T Limits: Tmin=0.860 Tmax=0.930
AbsCorr = MULTI-SCAN

Data completeness= 0.997          Theta(max)= 28.320

R(reflections)= 0.0251( 9657)     wR2(reflections)= 0.0658( 10839)

S = 1.047                   Npar= 433
```

The following ALERTS were generated. Each ALERT has the format
test-name_ALERT_alert-type_alert-level.
Click on the hyperlinks for more details of the test.

Alert level C

```
PLAT220_ALERT_2_C Non-Solvent  Resd 1  C   Ueq(max)/Ueq(min) Range        3.6 Ratio
PLAT241_ALERT_2_C High   'MainMol' Ueq as Compared to Neighbors of         N5 Check
PLAT242_ALERT_2_C Low    'MainMol' Ueq as Compared to Neighbors of        Si1 Check
PLAT242_ALERT_2_C Low    'MainMol' Ueq as Compared to Neighbors of        Si4 Check
PLAT413_ALERT_2_C Short Inter XH3 .. XHn     H9A      ..H21B     .       2.10 Ang.
                                                   -1+x,y,z  =      1_455 Check
PLAT910_ALERT_3_C Missing # of FCF Reflection(s) Below Theta(Min).          6 Note
```

Alert level G

```
PLAT232_ALERT_2_G Hirshfeld Test Diff (M-X)  Mo1      --N4       .        8.4 s.u.
PLAT380_ALERT_4_G Incorrectly? Oriented X(sp2)-Methyl Moiety .....        C29 Check
PLAT794_ALERT_5_G Tentative Bond Valency for Mo1       (V)       .       5.12 Info
PLAT912_ALERT_4_G Missing # of FCF Reflections Above STh/L=  0.600         21 Note
PLAT913_ALERT_3_G Missing # of Very Strong Reflections in FCF ....          1 Note
PLAT978_ALERT_2_G Number C-C Bonds with Positive Residual Density.          9 Info
```

0 **ALERT level A** = Most likely a serious problem - resolve or explain
0 **ALERT level B** = A potentially serious problem, consider carefully
6 **ALERT level C** = Check. Ensure it is not caused by an omission or oversight
6 **ALERT level G** = General information/check it is not something unexpected

0 ALERT type 1 CIF construction/syntax error, inconsistent or missing data
7 ALERT type 2 Indicator that the structure model may be wrong or deficient
2 ALERT type 3 Indicator that the structure quality may be low
2 ALERT type 4 Improvement, methodology, query or suggestion
1 ALERT type 5 Informative message, check

It is advisable to attempt to resolve as many as possible of the alerts in all categories. Often the minor alerts point to easily fixed oversights, errors and omissions in your CIF or refinement strategy, so attention to these fine details can be worthwhile. In order to resolve some of the more serious problems it may be necessary to carry out additional measurements or structure refinements. However, the purpose of your study may justify the reported deviations and the more serious of these should normally be commented upon in the discussion or experimental section of a paper or in the "special_details" fields of the CIF. checkCIF was carefully designed to identify outliers and unusual parameters, but every test has its limitations and alerts that are not important in a particular case may appear. Conversely, the absence of alerts does not guarantee there are no aspects of the results needing attention. It is up to the individual to critically assess their own results and, if necessary, seek expert advice.

Publication of your CIF in IUCr journals

A basic structural check has been run on your CIF. These basic checks will be run on all CIFs submitted for publication in IUCr journals (*Acta Crystallographica*, *Journal of Applied Crystallography*, *Journal of Synchrotron Radiation*); however, if you intend to submit to *Acta Crystallographica Section C* or *E* or *IUCrData*, you should make sure that full publication checks are run on the final version of your CIF prior to submission.

Publication of your CIF in other journals

Please refer to the *Notes for Authors* of the relevant journal for any special instructions relating to CIF submission.

PLATON version of 07/08/2019; check.def file version of 30/07/2019

Datablock ZDD_04_03 - ellipsoid plot

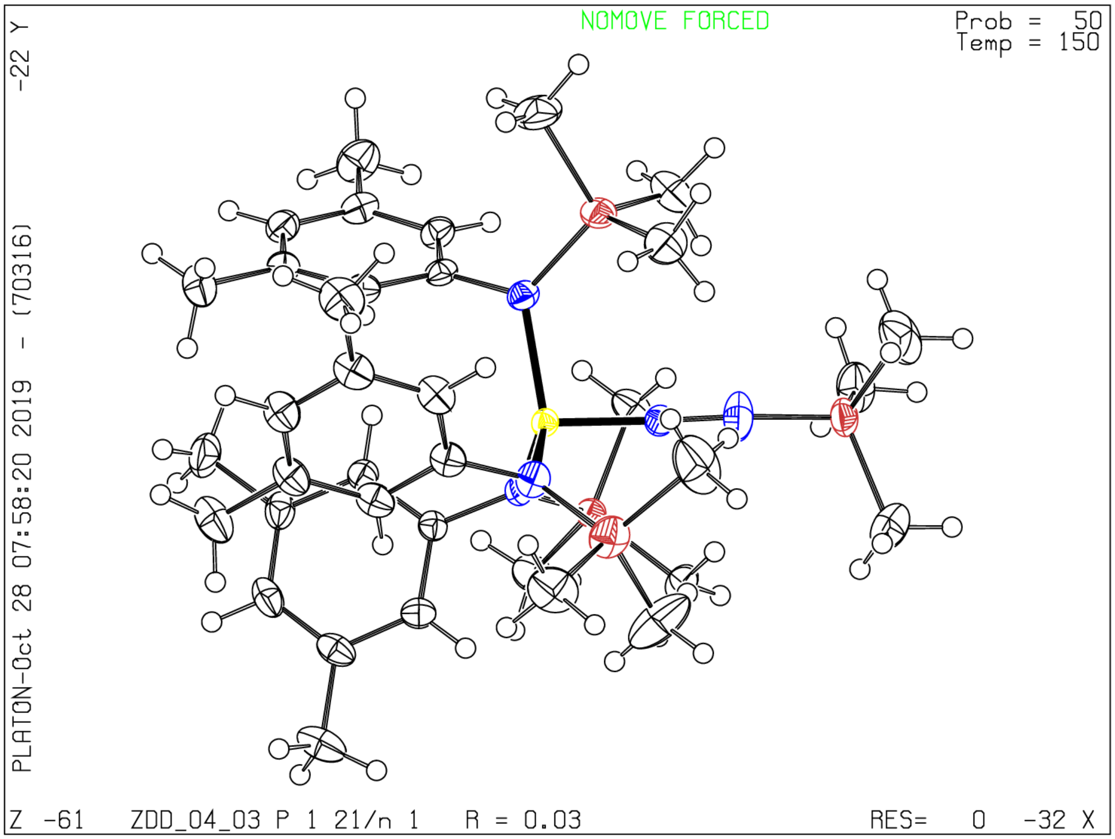

Supplement: nwaa290_Supplemental_Files [file nwaa290_supplemental_files.zip › Complex 6.pdf]
