# Supplementary material for: Silylamido supported dinitrogen heterobimetallic complexes: syntheses and their catalytic ability
Source: Natl Sci Rev. 2020 Dec 3;8(12):nwaa290. doi: 10.1093/nsr/nwaa290 (PMC8694672; doi:10.1093/nsr/nwaa290)

checkCIF/PLATON report

Structure factors have been supplied for datablock(s) zdd_03_53_2_0626

THIS REPORT IS FOR GUIDANCE ONLY. IF USED AS PART OF A REVIEW PROCEDURE FOR PUBLICATION, IT SHOULD NOT REPLACE THE EXPERTISE OF AN EXPERIENCED CRYSTALLOGRAPHIC REFEREE.

No syntax errors found. CIF dictionary Interpreting this report

Datablock: zdd_03_53_2_0626

```
Bond precision:  C-C = 0.0077 A         Wavelength=0.71073

Cell:            a=9.6898(7)     b=18.0835(13)      c=9.8507(6)
                 alpha=90        beta=99.818(2)     gamma=90
Temperature:     150 K
```

	Calculated	Reported
Volume	1700.8(2)	1700.8(2)
Space group	P 21	P 1 21 1
Hall group	P 2yb	P 2yb
Moiety formula	C30 H52 Mg N2 O2 Si2	C30 H52 Mg N2 O2 Si2
Sum formula	C30 H52 Mg N2 O2 Si2	C30 H52 Mg N2 O2 Si2
Mr	553.23	553.22
Dx,g cm-3	1.080	1.080
Z	2	2
Mu (mm-1)	0.149	0.149
F000	604.0	604.0
F000’	604.58	
h,k,lmax	12,24,13	13,24,13
Nref	8604[4434]	8503
Tmin,Tmax	0.968,0.974	0.920,0.970
Tmin’	0.953	

```
Correction method= # Reported T Limits: Tmin=0.920 Tmax=0.970
AbsCorr = MULTI-SCAN

Data completeness= 1.92/0.99      Theta(max)= 28.480

R(reflections)= 0.0611( 8137)     wR2(reflections)= 0.1716( 8503)

S = 1.064                  Npar= 354
```

The following ALERTS were generated. Each ALERT has the format
test-name_ALERT_alert-type_alert-level.
Click on the hyperlinks for more details of the test.

Alert level C

```
PLAT094_ALERT_2_C Ratio of Maximum / Minimum Residual Density ....       2.08 Report
PLAT213_ALERT_2_C Atom C29A            has ADP max/min Ratio .....       3.3 oblate
PLAT220_ALERT_2_C NonSolvent Resd 1  C   Ueq(max) / Ueq(min) Range       4.1 Ratio
PLAT241_ALERT_2_C High   'MainMol' Ueq as Compared to Neighbors of       C26 Check
PLAT241_ALERT_2_C High   'MainMol' Ueq as Compared to Neighbors of       C28 Check
PLAT242_ALERT_2_C Low    'MainMol' Ueq as Compared to Neighbors of       C27 Check
PLAT340_ALERT_3_C Low Bond Precision on  C-C Bonds ...............    0.0077 Ang.
PLAT910_ALERT_3_C Missing # of FCF Reflection(s) Below Theta(Min).         6 Note
PLAT911_ALERT_3_C Missing FCF Refl Between Thmin & STh/L=    0.600        14 Report
PLAT913_ALERT_3_C Missing # of Very Strong Reflections in FCF ....         8 Note
PLAT934_ALERT_3_C Number of (Iobs-Icalc)/Sigma(W) > 10 Outliers ..         1 Check
```

Alert level G

```
PLAT301_ALERT_3_G Main Residue  Disorder ..............(Resd  1  )        3% Note
PLAT773_ALERT_2_G Check long C-C Bond in CIF: C29      --C30            1.75 Ang.
PLAT779_ALERT_4_G Suspect or Irrelevant (Bond) Angle(s) in CIF . #        83 Check
              N2   -C12  -MG1     1.555   1.555   1.555              39.93 Deg.
PLAT912_ALERT_4_G Missing # of FCF Reflections Above STh/L=  0.600        21 Note
PLAT978_ALERT_2_G Number C-C Bonds with Positive Residual Density.         1 Info
PLAT992_ALERT_5_G Repd & Actual _reflns_number_gt Values Differ by         2 Check
```

0 **ALERT level A** = Most likely a serious problem - resolve or explain
0 **ALERT level B** = A potentially serious problem, consider carefully
11 **ALERT level C** = Check. Ensure it is not caused by an omission or oversight
6 **ALERT level G** = General information/check it is not something unexpected

0 ALERT type 1 CIF construction/syntax error, inconsistent or missing data
8 ALERT type 2 Indicator that the structure model may be wrong or deficient
6 ALERT type 3 Indicator that the structure quality may be low
2 ALERT type 4 Improvement, methodology, query or suggestion
1 ALERT type 5 Informative message, check

It is advisable to attempt to resolve as many as possible of the alerts in all categories. Often the minor alerts point to easily fixed oversights, errors and omissions in your CIF or refinement strategy, so attention to these fine details can be worthwhile. In order to resolve some of the more serious problems it may be necessary to carry out additional measurements or structure refinements. However, the purpose of your study may justify the reported deviations and the more serious of these should normally be commented upon in the discussion or experimental section of a paper or in the "special_details" fields of the CIF. checkCIF was carefully designed to identify outliers and unusual parameters, but every test has its limitations and alerts that are not important in a particular case may appear. Conversely, the absence of alerts does not guarantee there are no aspects of the results needing attention. It is up to the individual to critically assess their own results and, if necessary, seek expert advice.

Publication of your CIF in IUCr journals

A basic structural check has been run on your CIF. These basic checks will be run on all CIFs submitted for publication in IUCr journals (*Acta Crystallographica*, *Journal of Applied Crystallography*, *Journal of Synchrotron Radiation*); however, if you intend to submit to *Acta Crystallographica Section C* or *E* or *IUCrData*, you should make sure that full publication checks are run on the final version of your CIF prior to submission.

Publication of your CIF in other journals

Please refer to the *Notes for Authors* of the relevant journal for any special instructions relating to CIF submission.

PLATON version of 04/06/2020; check.def file version of 02/06/2020

Datablock zdd_03_53_2_0626 - ellipsoid plot

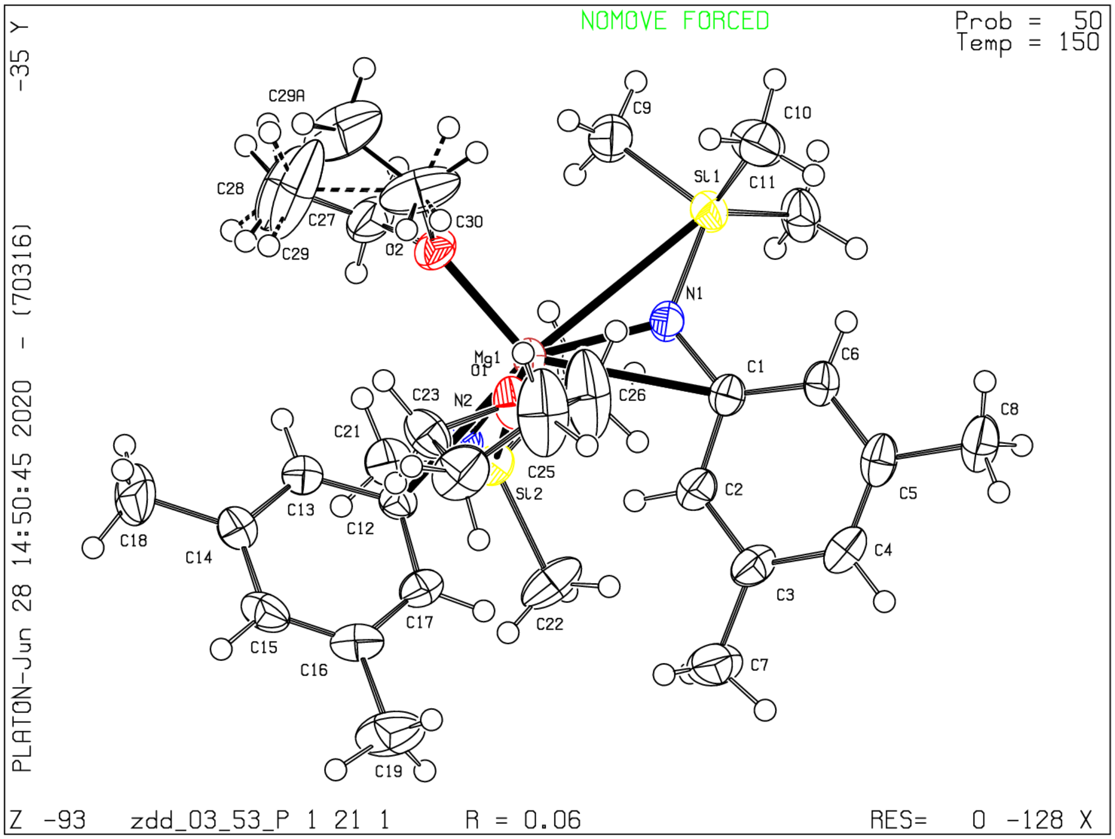

Supplement: nwaa290_Supplemental_Files [file nwaa290_supplemental_files.zip › Mg[N(SiMe3)Ar]2.pdf]
