# Supplementary material for: Silylamido supported dinitrogen heterobimetallic complexes: syntheses and their catalytic ability
Source: Natl Sci Rev. 2020 Dec 3;8(12):nwaa290. doi: 10.1093/nsr/nwaa290 (PMC8694672; doi:10.1093/nsr/nwaa290)
Supplement: nwaa290_Supplemental_Files [file nwaa290_supplemental_files.zip › SI_revised_final.pdf]

Supporting Information

Silylamido Supported Dinitrogen Heterobimetallic Complexes: Syntheses and Their Catalytic Ability

Dan-dan Zhai [a], Si-jun Xie [a], Yi Xia [a], Hua-yi Fang [a,*] and Zhang-jie Shi [a, b,*]

[a] *Department of Chemistry, Fudan University, Shanghai 200433*

[b] *State Key Laboratory of Organometallic Chemistry, Shanghai Institute of Organic Chemistry, Chinese Academy of Sciences, Shanghai 200032, China*

*To whom correspondence should be addressed. E-mail address: zjshi@fudan.edu.cn (Z. Shi); zshi@pku.edu.cn (Z. Shi); hfang@fudan.edu.cn (H. Fang)

Contents

1. General Information 2
2. Synthesis and Characterization of Molybdenum Complexes 3
3. Disproportionation of Cyclohexadienes 7
4. Isomerization of Terminal Alkenes Catalyzed by Complex **5** 19
5. Mechanistic Studies 21
6. NMR Data 25
7. References 30

1. General Information

All manipulations of air- and moisture-sensitive compounds were performed under an argon/nitrogen atmosphere by use of standard Schlenk techniques and in Vigor gloveboxes. Et_2O, THF, hexane, pentane and C_6D_6 were dried by distillation over sodium/benzophenone. Olefins (1,3-cyclohexadiene, 1,4-cyclohexadiene, allylbenzene and 1-hexene) were purchased from TCI and J&K, dried over CaH_2, degassed and kept in a N_2 glovebox prior to use. Argon gas (99.999%) and Nitrogen gas (99.999%) were purchased from Dumaoai Gas Co., Ltd, Shanghai. Compounds $MoCl_3(THF)_3$ **1** [1], $HN(SiMe_3)Ar$ (Ar = 3,5-$Me_2C_6H_3$) [2], $LiN(SiMe_3)Ar$ (**2**, Ar = 3,5-$Me_2C_6H_3$) [2], **16** [3-4], **16-*d₁*** [3-4], **16-*d₂*** [3-4] were prepared according to the procedures described in the literatures. $Mg[N(SiMe_3)Ar]_2$ (Ar = 3,5-$Me_2C_6H_3$) was prepared from the recation of Mg(*n*-Bu)$_2$ and 2 equiv of $HN(SiMe_3)Ar$ in the solution of Hexane/Et_2O (5/1). ^1H NMR, ^{13}C NMR and inverse gated decoupled ^{13}C NMR spectra were recorded on a Bruker AVANCE 400 MHz instrument (400 MHz for ^1H, 101 MHz for ^{13}C). Elemental analysis was performed by the Analytical Laboratory of Shanghai Institute of Organic Chemistry, Chinese Academy of Sciences. IR spectra of solid samples were recorded with a NICOLET AVATAR 330 FT-IR spectrophotometer on KBr pallets.

X-ray Crystallographic Studies

Data collections for complex **3, 4, 5, 6** and $Mg(THF)_2[N(SiMe_3)Ar]_2$ were performed on a Bruker D8 Venture Photon II using Mo-Kα radiation (λ = 0.71073 Å). Their structure were solved by direct methods and refined by full-matrix least squares on F^2. All non-hydrogen atoms were refined anisotropically. The Diamond program was utilized to draw the molecules. CCDC 1963795, 1963701, 1963702, 1963703, and 1963706 contain the supplementary crystallographic data for this paper. These data can be obtained free of charge from The Cambridge Crystallographic Data Centre via www.ccdc.cam.ac.uk/data_request/cif.

2. Synthesis and Characterization of Molybdenum Complexes

2.1 Synthesis of Mo[N(SiMe$_3$)Ar]$_3$ (3)

In a standard Schlenk line of Ar atmosphere, 30 mL of ether was added into the mixture of MoCl$_3$(THF)$_3$ (1.05 g, 2.51 mmol) and LiN(SiMe$_3$)Ar (0.75 g, 3.77 mmol) at -78 ˚C with an acetone/ liquid nitrogen bath, then the reaction was kept at room temperature for 5 h. After filtration to remove a pale orange precipitate, the resulting ether solution was concentrated and stored in an Ar glovebox refrigerator to afford red crystalline complex **3** (0.52 g, 0.77 mmol, 61%). ^{1}H NMR (400 MHz, C$_6$D$_6$): δ 45.75 (br s, 27H, Si(CH_3)$_3$), 2.19 (br s, 6H, ortho Ar*H*), -2.22 (br s, 18H, ArCH_3), -30.53 (br s, 3H, paran Ar*H*). The product contained small amounts (< 2 % based on ^{1}H NMR integration) of *N*-(Trimethylsilyl)-3,5-dimethylaniline. Anal. Calcd for $C_{33}H_{54}MoN_3Si_3$: C, 58.89; H, 8.09; N, 6.24. Found: C, 58.83; H, 8.07; N, 6.37.

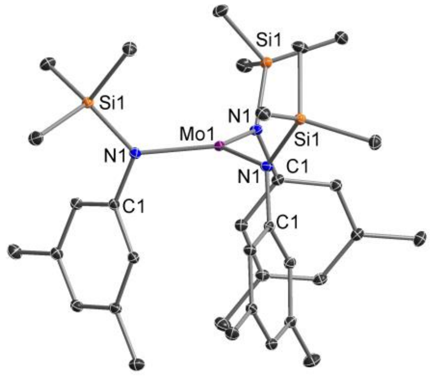

Figure S1. Crystal structure of complex **3** with 10% thermal ellipsoids. H atoms have been omitted for clarity. Selected bond distances (Å) and angles (deg): Mo1-N1, 1.9854(16); N1-Si1, 1.7507(15); N1-Mo1-N1, 119.755(6); Mo1-N1-Si1, 126.306(112); Mo1-N1-C1, 113.316(115); C1-N1-Si1, 120.295(198).

2.2 Synthesis of ClMo[N(SiMe$_3$)Ar]$_3$ (4)

In a standard Schlenk line of Ar atmosphere, 40 mL of ether was added into the mixture of MoCl$_3$(THF)$_3$ (1.50 g, 3.58 mmol) and LiN(SiMe$_3$)Ar (1.40 g, 7.17 mmol) at -78 ˚C with an acetone/ liquid nitrogen bath, then the reaction was kept at room temperature for 48 h. After filtration to remove a dark amber precipitate, the resulting ether solution was concentrated and stored in an Ar glovebox refrigerator to afford red solid (0.98 g, 1.38 mmol, 58%). ^{1}H NMR (400 MHz, C$_6$D$_6$): δ 10.91 (br s, 27H, Si(CH_3)$_3$), 7.69 (br s, 6H, ortho Ar*H*), 4.21 (br s, 3H, para Ar*H*), 2.56 (br s, 18H, ArCH_3). ^{13}C NMR (101 MHz, C$_6$D$_6$): 227.85, 166.68, 145.61, 64.79, 29.08. The product contained small amounts (< 2 % based on ^{1}H NMR integration) of *N*-(Trimethylsilyl)-3,5-dimethylaniline. Anal. Calcd for $C_{33}H_{54}ClMoN_3Si_3$: C, 55.95; H, 7.68; N, 5.93. Found: C, 55.13; H, 7.62; N, 5.85.

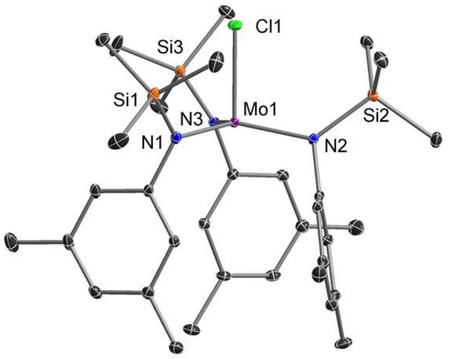

Figure S2. Crystal structure of complex **4** with 10% thermal ellipsoids. H atoms have been omitted for clarity. Selected bond distances (Å) and angles (deg): Mo1-N1, 1.9576(40); Mo1-N2, 1.9635(36); Mo1-N3, 1.9618(38); Mo1-Cl1, 2.3221(16); N1-Mo1-N2, 119.055(160); N2-Mo1-N3, 116.802(154); N3-Mo1-N1, 119.131(164); Cl1-Mo1-N1, 95.977(127); Cl1-Mo1-N2, 99.591(119); Cl1-Mo1-N3, 96.914(114).

2.3 Synthesis of [Ar(Me_3Si)N]$_3$MoN_2Mg(THF)$_2$[N($SiMe_3$)Ar] (5)

In a standard Schlenk line of Ar atmosphere, 40 mL of ether was added into the mixture of $MoCl_3$(THF)$_3$ (2.00 g, 4.78 mmol) and LiN($SiMe_3$)Ar (1.90 g, 9.55 mmol) at -78 ˚C with an acetone/ liquid nitrogen bath, then the reaction was kept at room temperature for 48 h. After filtration, volatile material was removed under reduced pressure to provide a black-red solid. The solid was transferred to a N_2 glovebox and dissolved in THF (20 mL). To the solution was added Mg powder (348.6 mg, 14.34 mmol) and stirred at room temperature for 24 h. After solvent removal in vacuo, the residue was extracted with ether. The resulting ether solution was concentrated and stored in the N_2 glovebox refrigerator to afford red solid (0.96 g, 0.90 mmol, 28%). ^{1}H NMR (400 MHz, C_6D_6): δ 6.77 (s, 2H), 6.58 (s, 3H), 6.48 (s, 1H), 6.19 (s, 6H), 3.64 (m, 8H), 2.31 (s, 6H), 2.10 (s, 18H), 1.26 (m, 8H), 0.61 (s, 27H), 0.48 (s, 9H). ^{13}C NMR (101 MHz, C_6D_6): 158.88, 153.44, 137.65, 127.35, 125.62, 124.70, 119.80, 70.13, 30.22, 25.04, 21.79, 21.61, 4.16, 3.27. IR: ν_{NN} (KBr) = 1596.8 cm^{-1}. A satisfied elemental analysis for **5** was not obtained. A little amount of impurity was observed in the ^{1}H NMR spectrum of the powder form even though pure crystals could be collected from ether solution; the following are typical results. Anal. Calcd for $C_{52}H_{88}MgMoN_6O_2Si_4$: C, 58.82; H, 8.35; N, 7.91. Found: C, 57.55; H, 8.30; N, 7.47. Attempted to extract with toluene and recrystallize in the glovebox refrigerator afforded red solid. Found: C, 58.04; H, 8.43; N, 7.55.

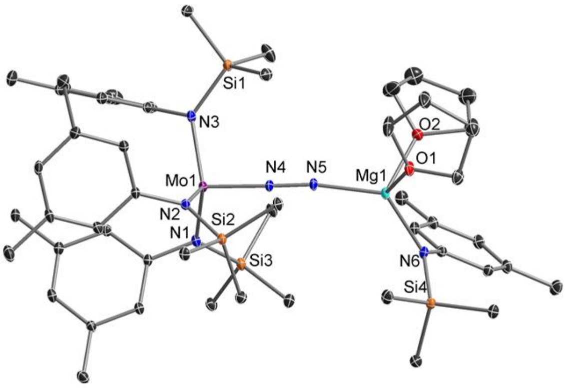

Figure S3. Crystal structure of complex **5** with 10% thermal ellipsoids. H atoms have been omitted for clarity. Selected bond distances (Å) and angles (deg): Mo1-N1, 1.998(4); Mo1-N2, 2.0066(34); Mo1-N3, 2.0150(39); Mo1-N4, 1.8110(34); N4-N5, 1.1942(48); Mg1-N5,1.9621(35); Mg1-N6,1.9924(31); Mo1-N4-N5, 178.107(287); N4-N5-Mg1, 171.026(282).

2.4 Synthesis of [Ar(Me_3Si)N]$_3$MoN_2SiMe_3 (6)

In a standard Schlenk line of Ar atmosphere, the fresh distilled Me_3SiCl (153.2 mg, 1.41 mmol) in ether (10 mL) was added to a solution of complex **5** (0.50 g, 0.47 mmol) in ether (15 mL) at -78 ˚C with an acetone/liquid nitrogen bath, then the reaction was kept at room temperature for 5 h. After filtration through Celite, volatile material was removed under reduced pressure. The resulting resuide was recrystallized from pentane to afford yellow crystalline **6** (0.28 g, 0.36 mmol, 77%).^{1}H NMR (400 MHz, C_6D_6): δ 6.55 (s, 3H), 6.06 (s, 6H), 2.02 (s, 18H), 0.47 (s, 9H), 0.45 (s, 27H). ^{13}C NMR (101 MHz, C_6D_6): δ 150.20, 138.23, 127.46, 126.91, 21.45, 3.31, 3.15. IR: ν_{NN} (KBr) = 1597.4 cm^{-1}. Anal. Calcd for $C_{36}H_{63}MoN_5Si_4$: C, 55.85; H, 8.20; N, 9.05. Found: C, 55.92; H, 8.28; N, 8.88.

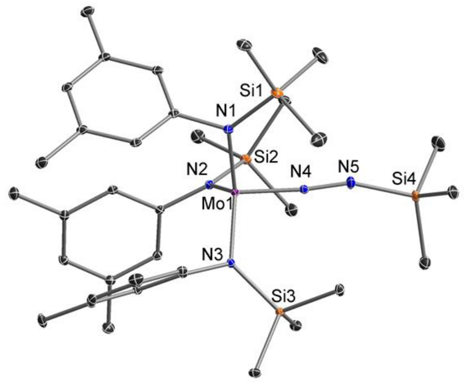

Figure S4. Crystal structure of complex **6** with 10% thermal ellipsoids. H atoms have been omitted for clarity. Selected bond distances (Å) and angles (deg): Mo1-N1, 1.9815(14); Mo1-N2, 1.9911(12); Mo1-N3, 1.9785(12); Mo1-N4, 1.7707(12); N4-N5, 1.2139(18); Si4-N5,1.7092(16); Mo1-N4-N5, 174.306(118); N4-N5-Si4, 160.513(136).

2.5 Attempted synthesis of ClMo[N(SiMe$_2$^{t}Bu)Ar]$_3$

In a standard Schlenk line of Ar atmosphere, 30 mL of ether was added into the mixture of $MoCl_3(THF)_3$ (800 mg, 1.91 mmol) and LiN(SiMe$_2$^{t}Bu)Ar (922 mg, 3.82 mmol) at -78 ˚C with an acetone/ liquid nitrogen bath, then the reaction was kept at room temperature for 20 h. After filtration to remove a dark amber precipitate, the resulting ether solution was concentrated and stored in a Ar glovebox refrigerator to afford purple solid (476 mg, 0.57 mmol, 44%). ^{1}H NMR (400 MHz, C_6D_6): δ10.43 (br), 4.93 (br), 2.92 (br), 1.56 (br). Single crystals of the title complex suitable for X-ray diffraction study were grown solution of ether at -35 ˚C (**Figure S5**, CCDC-2010825 contained the supplementary crystallographic data for this paper). However, the structure suffered from disorders of the -N(SiMe$_2$^{t}Bu) substituent. A little amount of free ligand HN(SiMe$_2$^{t}Bu)Ar was observed in the ^{1}H NMR spectrum of the complex and satisfied elemental analysis was not obtained.

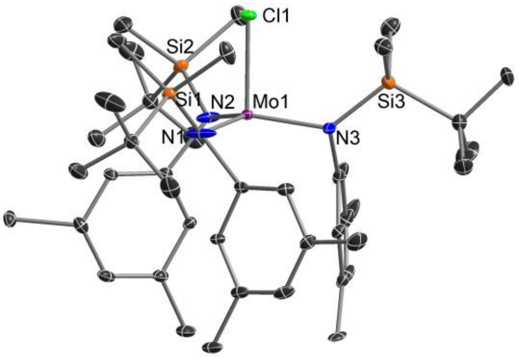

Figure S5. Crystal structure of complex ClMo[N(SiMe$_2$^{t}Bu)Ar]$_3$ with 10% thermal ellipsoids. H atoms have been omitted for clarity. Selected bond distances (Å) and angles (deg): Mo1-N1, 1.9339(61); Mo1-N2, 1.9407(39); Mo1-N3, 1.9323(57); Mo1-Cl1, 2.3258(11); N1-Mo1-N2, 116.978(185); N2-Mo1-N3, 119.955(237); N3-Mo1-N1, 117.194(278); Cl1-Mo1-N1, 97.681(134); Cl1-Mo1-N2, 98.438(103); Cl1-Mo1-N3, 98.200(137).

2.6 Attempted synthesis of [Ar(^{t}BuMe$_2$Si)N]$_3$MoN$_2$Mg(THF)$_2$[N(SiMe$_2$^{t}Bu)Ar]

In a N$_2$ glovebox, Mg powder (33.9 mg, 1.40 mmol) was added to a solution of complex ClMo[N(SiMe$_2$^{t}Bu)Ar]$_3$ (390 mg, 0.46 mmol) in THF (15 mL) at room temperature and stirred for 24 h. After that, solvents were removed under reduced pressure and the resuide was extracted by ether. The resulting ether solution was concentrated and stored in the N$_2$ glovebox refrigerator to afford red solid (205 mg, 0.17 mmol, 36%). ^{1}H NMR (400 MHz, C$_6$D$_6$): δ 6.83 (s, 2H), 6.62 (s, 3H), 6.46 (s, 1H), 6.03 (s, 6H), 3.64 (m, 12H), 2.29 (s, 6H), 2.12 (s, 18H), 1.32 (m, 12H), 1.14 (s, 9H), 0.99 (s, 27H), 0.78 (s, 18H), 0.50 (s, 6H). Single crystals of the title copmplex suitable for X-ray diffraction study were grown solution of ether at -35 ˚C (**Figure S6**, CCDC-2010829 contained the supplementary crystallographic data for this paper). However, only small amount of solid could be recrystallized from solution of ether in many repeated experiments and a little amount of impurity was observed in the NMR spectrum. Attempts to obtain analytically pure material failed.

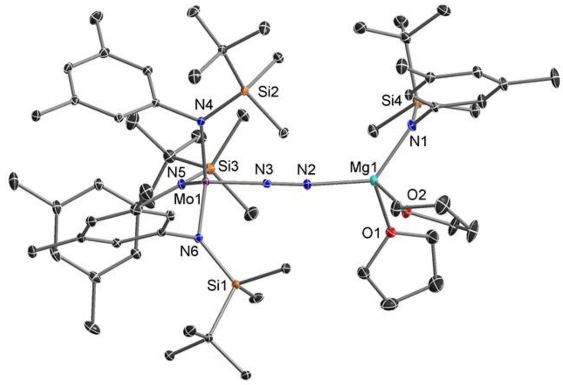

Figure S6. Crystal structure of complex [Ar(^{t}BuMe$_2$Si)N]$_3$MoN$_2$Mg(THF)$_2$[N(SiMe$_2$^{t}Bu)Ar] with 10% thermal ellipsoids. H atoms have been omitted for clarity. Selected bond distances (Å) and angles (deg): Mo1-N4, 2.0060(21); Mo1-N5, 2.0032(20); Mo1-N6, 2.0055(22); Mo1-N3, 1.8095(20); N3-N2, 1.1988(30); Mg1-N2, 1.9825(24); Mg1-N1, 1.9993(25); Mo1-N3-N2, 179.685(197); N3-N2-Mg1, 174.897(201).

3. Disproportionation of Cyclohexadienes

3.1 Stoichiometric reaction of 1,3-Cyclohexadiene (7) with complex 5

In a N_2 glovebox, to a J. Young valve NMR tube were added complex **5** (10.6 mg, 0.01 mmol), C_6D_6 (0.5 mL) and 1,3-Cyclohexadiene (**7**) (0.50 mmol in 500 μL C_6D_6, 10 μL, 0.01 mmol). The tube was heated at 100 °C for 24 h. The ^{1}H NMR analysis revealed that **7** transformed completely to produce benzene (**8**) and cyclohexene (**9**), meanwhile featured signals of **5** remained in the ^{1}H NMR spectrum of reaction mixture (**Figure S7, b**). Trace of cyclohexane (**10**) was observed in the reaction. The products were identified by comparing corresponding ^{1}H NMR spectrum with commercial samples.

Benzene (8) ^{1}H NMR (400 MHz, C_6D_6): δ 7.16 (s, H, *CH*).

Cyclohexene (9) ^{1}H NMR (400 MHz, C_6D_6): δ 5.70 (s, 2H, *CH*CH_2CH_2), 1.91 (m, 4H, CH*CH_2*CH_2), 1.52 (m, 4H, $CHCH_2$*CH_2*).

Cyclohexane (10) ^{1}H NMR (400 MHz, C_6D_6): δ 1.41 (s, CH_2).

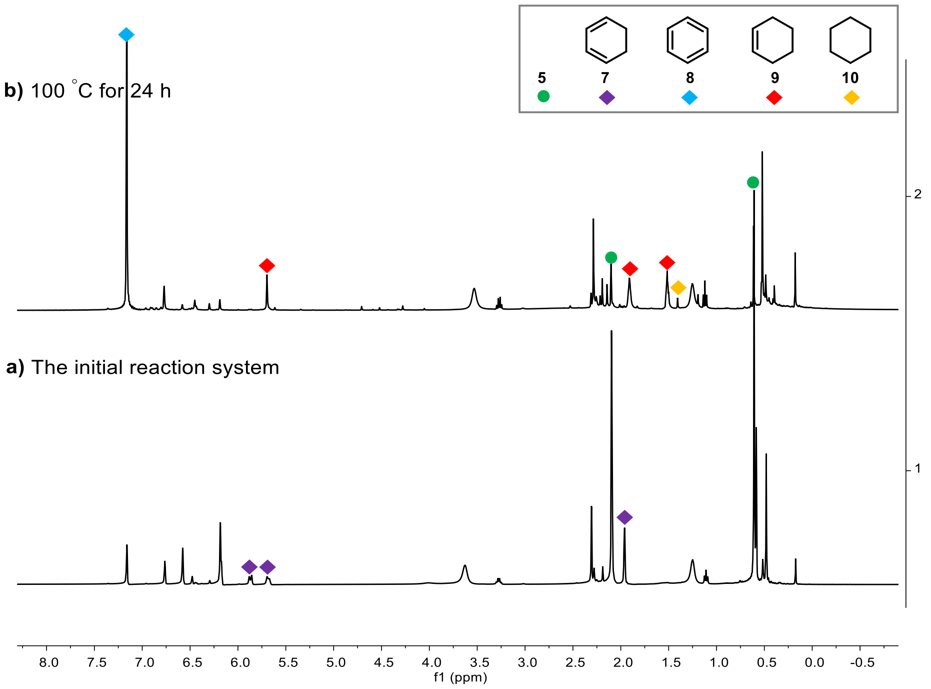

Figure S7. Stoichiometric reaction of **7** with complex **5**

3.2 Disproportionation of 1,3-cyclohexadiene (7) catalyzed by complex 5

(1) ***Stepwise method*:** In a N_2 glovebox, to the J. Young valve NMR tube (the one above) was added **7** (16.0 × 3 mg, 0.20 × 3 mmol) in three portion. The tube was heated at 100 °C and monitored by NMR. The ^{1}H NMR analysis revealed that **7** could be catalyzed by complex **5** to produce **8** and **9** smoothly with 90% conversion after 444 h (**Figure S8**, catalyst/substrate = 0.016/1). 1,4-cyclohexadiene (**11**) and trace of **10** were observed in the reaction, which were identified by comparing corresponding ^{1}H NMR spectrum with commercial samples. Conversion values were obtained from the relative integrations of **7** (δ 5.88~5.86, *CH*$CHCH_2$) and **9** (δ 1.57~1.47, $CHCH_2$*CH_2*).

1,4-Cyclohexadiene (**11**) ^{1}H NMR (400 MHz, C_6D_6): δ 5.61 (s, 4H, *CH*CH_2), 2.53 (s, 4H, CH*CH_2*).

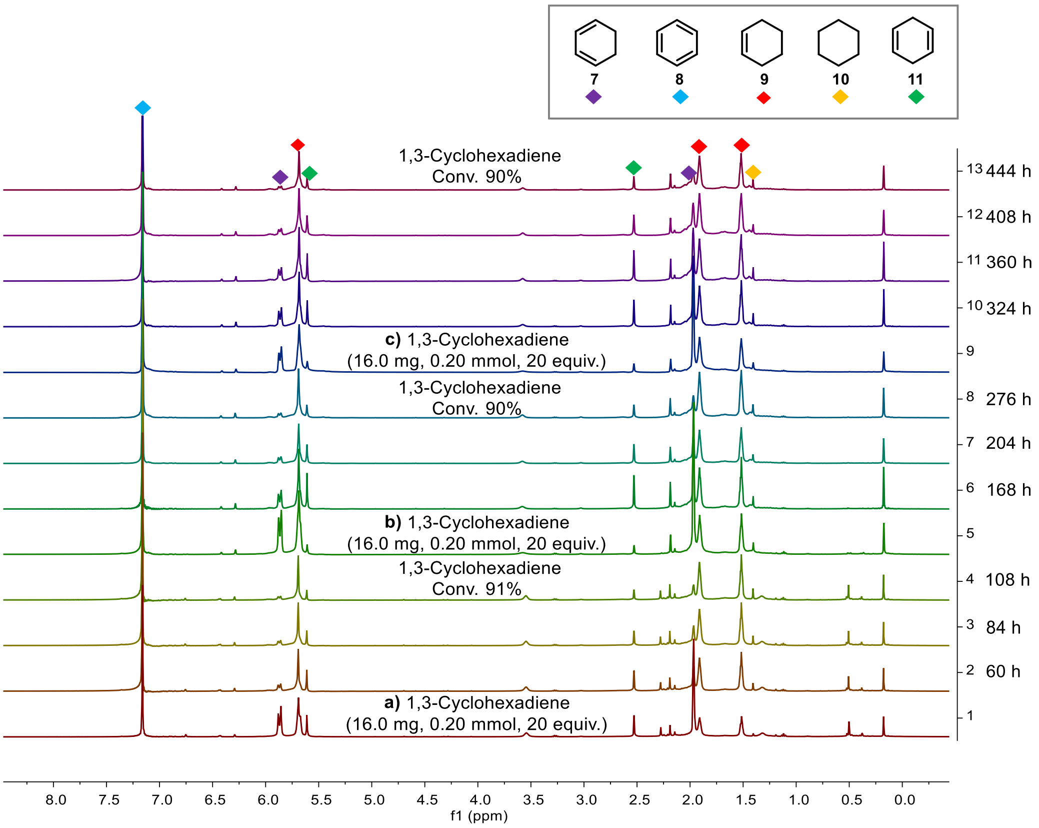

Figure S8. Stepwise disproportionation of **7** catalyzed by complex **5**

(2) ***One-pot method*** (catalyst/substrate = 0.017/1): In a N_2 glovebox, to a J. Young valve NMR tube was added complex **5** (10.6 mg, 0.01 mmol), C_6D_6 (0.5 mL) and **7** (48.1 mg, 0.60 mmol). The tube was heated at 100 °C and monitored by NMR. The ^{1}H NMR analysis revealed that **7** was catalyzed by complex **5** to produce **8** and **9** smoothly with 99% conversion after 180 h (**Figure S9**).

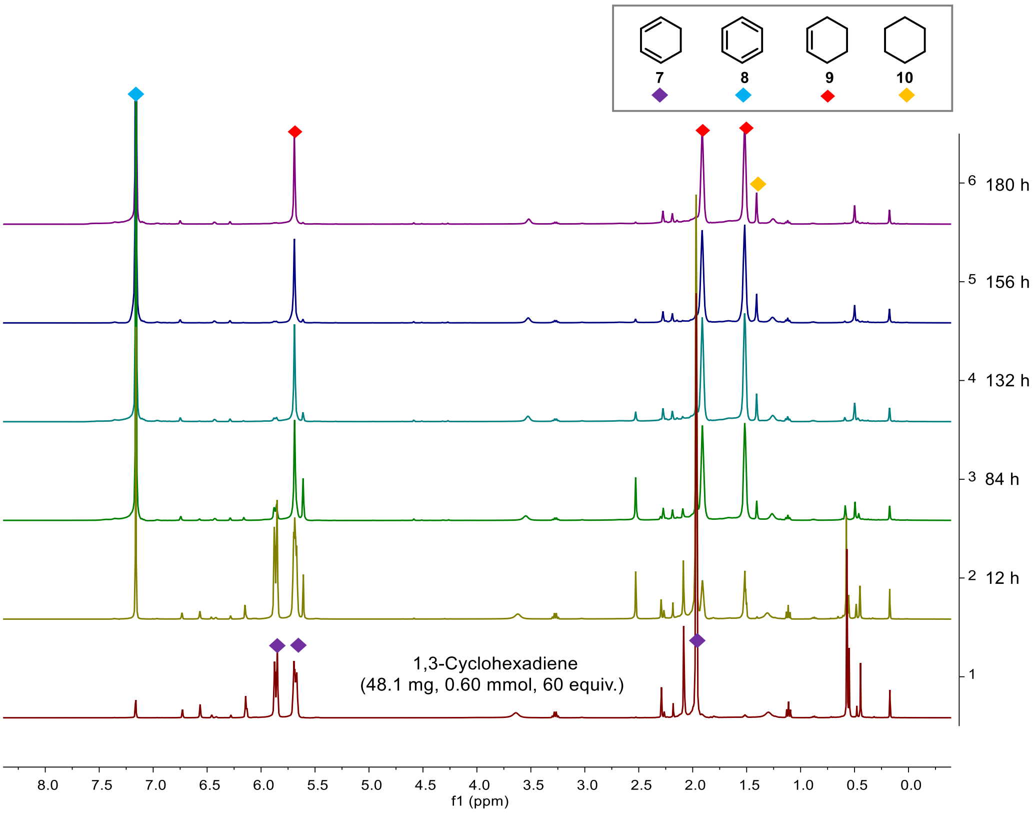

Figure S9. One-pot disproportionation of **7** catalyzed by complex **5** (catalyst/substrate = 0.017/1)

(3) ***One-pot method*** (catalyst/substrate = 0.10/1): In a N_2 glovebox, to a J. Young valve NMR tube was added complex **5** (31.8 mg, 0.03 mmol), C_6D_6 (0.5 mL) and **7** (24.0 mg, 0.30 mmol). The tube was heated at 100 °C and monitored by NMR. The ^{1}H NMR analysis revealed that the conversion of **7** was 99% after 24 h (**Figure S10, b**).

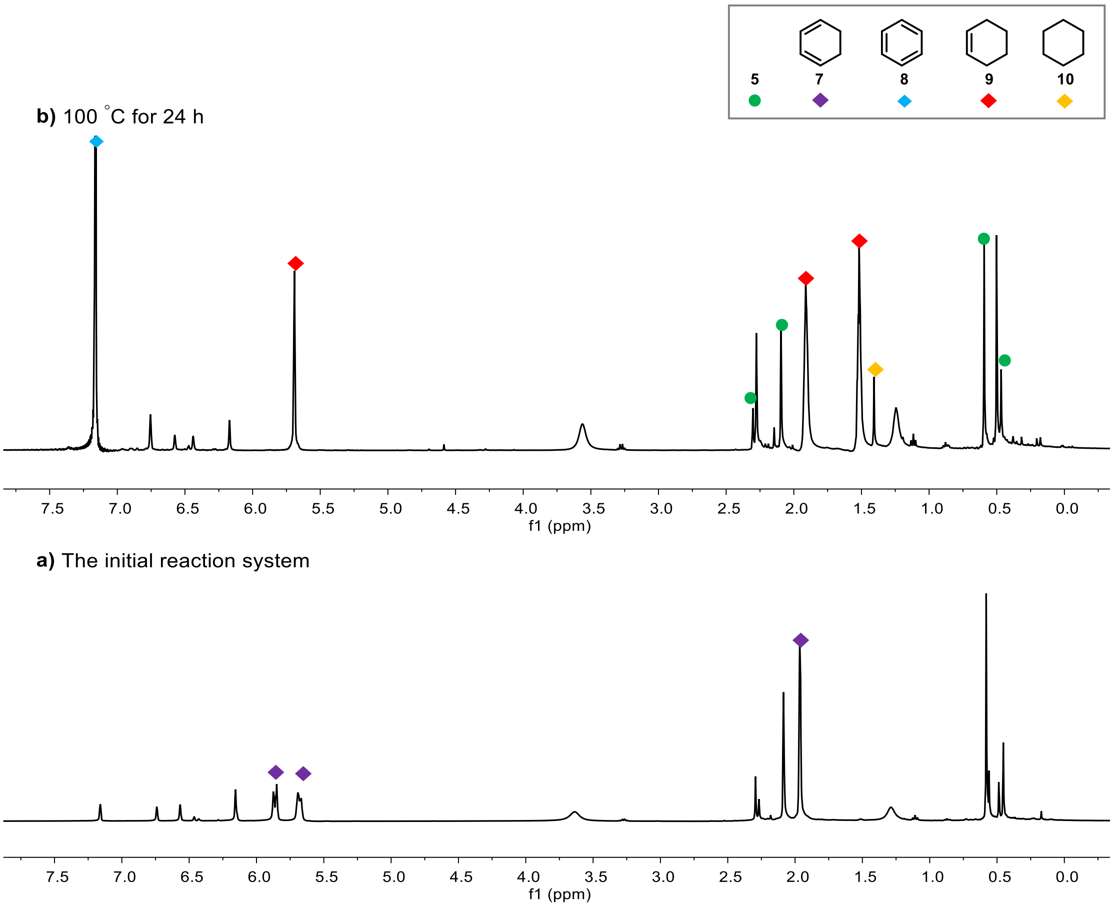

Figure S10. Disproportionation of **7** catalyzed by complex **5** (catalyst/substrate = 0.10/1)

(4) ***Under Ar atmosphere*****:** In an Ar glovebox, to a J. Young valve NMR tube was added complex **5** (31.8 mg, 0.03 mmol), C_6D_6 (0.5 mL) and **7** (24.0 mg, 0.30 mmol). The tube was heated at 100 °C and monitored by NMR. The ^1H NMR analysis revealed that the conversion of **7** was 98% after 24 h (**Figure S11, b**). Conversion values were obtained from the relative integrations of **7** (δ 5.88~5.86, *CH*$CHCH_2$) and **9** (δ 1.57~1.47, $CHCH_2$*CH_2*).

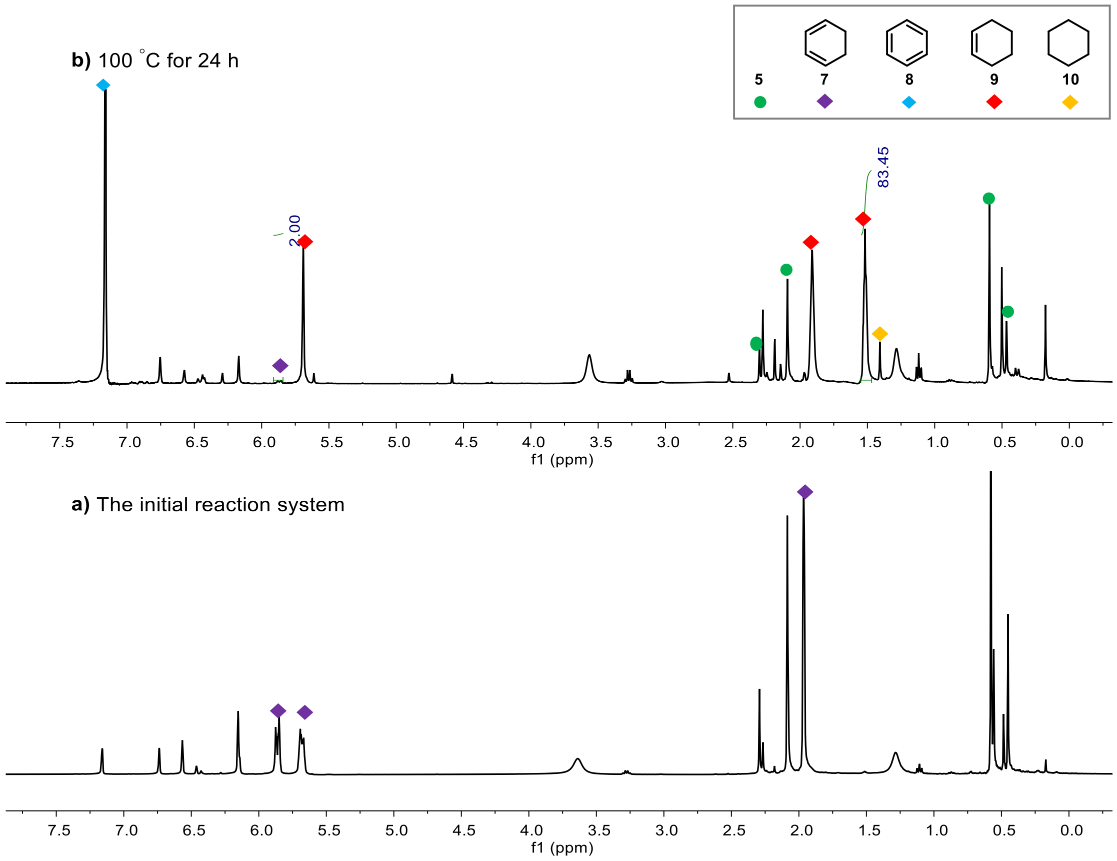

Figure S11. Disproportionation of **7** catalyzed by complex **5** under Ar atomsphere

(5) ***Under 10 atm of N_2*:** In a N_2 glovebox, to an oven-dried vessel was added a C_6D_6 solution (0.5 mL) of complex **5** (31.8 mg, 0.03 mmol) and **7** (24.0 mg, 0.30 mmol). The vessel was then placed into an autoclave, pressurized with 10 atm of N_2 and stirred at 100 °C for 24 h. The ^{1}H NMR analysis revealed that the conversion of **7** was 99% after 24 h (**Figure S12**).

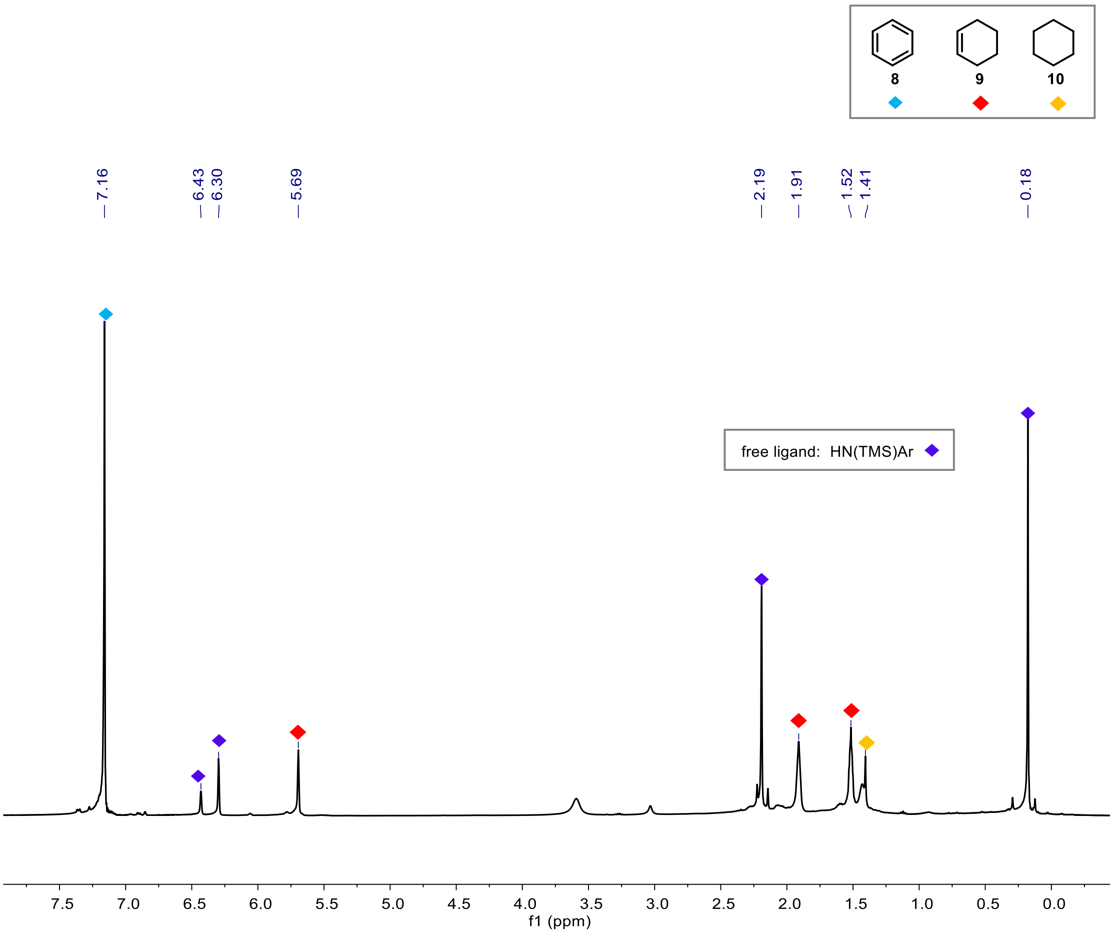

Figure S12. Disproportionation of **7** catalyzed by complex **5** under 10 atm of N_2

3.3 Disproportionation of 1,4-cyclohexadiene (11) catalyzed by complex 5

In a N_2 glovebox, to a J. Young valve NMR tube was added complex **5** (31.8 mg, 0.03 mmol), C_6D_6 (0.5 mL), naphthalene (12.8 mg, 0.10 mmol, as internal standard) and **11** (24.0 mg, 0.30 mmol). The tube was heated at 100 °C and monitored by NMR. The ^{1}H NMR analysis revealed that **11** was catalyzed by complex **5** to produce **8** and **9** smoothly (**Figure S13**). **7** and trace of **10** were observed during the reaction. The conversion of **11** was 99% after 48 h, meanwhile the isomerized **7** was < 1%.

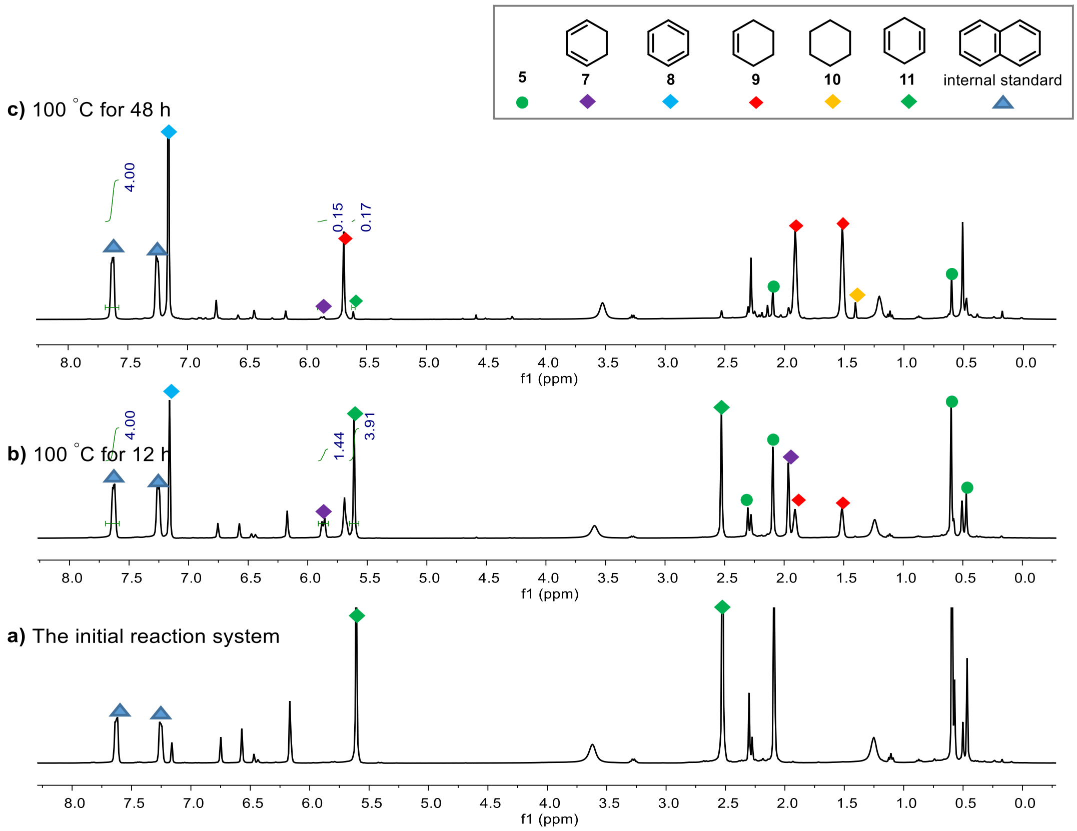

Figure S13. Disproportionation of **11** catalyzed by complex **5**

3.4 Effects of disproportionation of 1,3-cyclohexadiene (7) catalyzed by other complexes

(1) Complex **6** as catalyst

In a N_2 glovebox, to a J. Young valve NMR tube was added complex **6** (23.2 mg, 0.03 mmol), C_6D_6 (0.5 mL), naphthalene (12.8 mg, 0.10 mmol, as internal standard) and **7** (24.0 mg, 0.30 mmol). The tube was heated at 100 °C. The ^{1}H NMR analysis revealed that the conversion of **7** was 94% and 98% respectively after 12 h and 20 h (**Figure S14, b** and **c**).

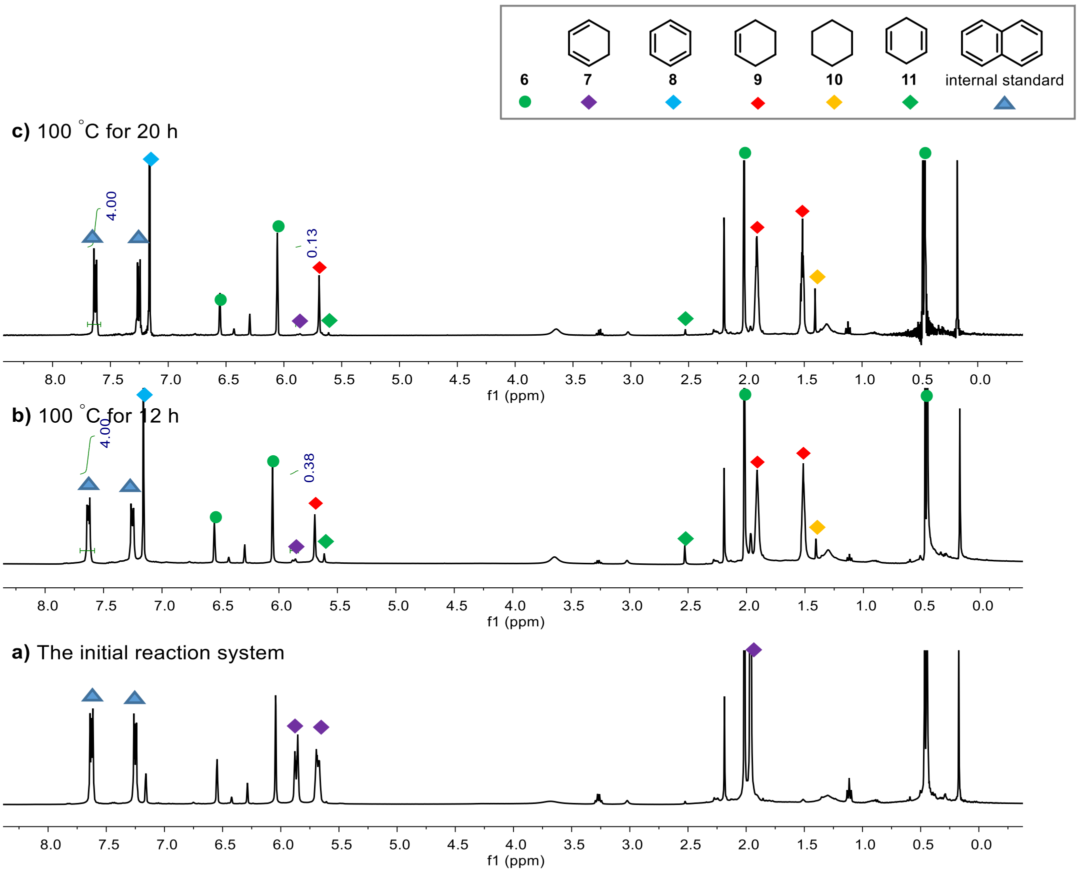

Figure S14. Disproportionation of **7** catalyzed by complex **6**

(2) Complex **3** as catalyst

In a N_2 glovebox, to a J. Young valve NMR tube was added complex **3** (20.5 mg, 0.03 mmol), C_6D_6 (0.5 mL) and **7** (24.0 mg, 0.30 mmol). The tube was heated at 100 °C for 24 h. The ^{1}H NMR analysis revealed that the conversion of **7** was 15% (**Figure S15**, **c**). It should be noted that the featured signals of **3** could not be observed in the reaction after heating at 100 °C for 5 h (**Figure S16**, **b**).

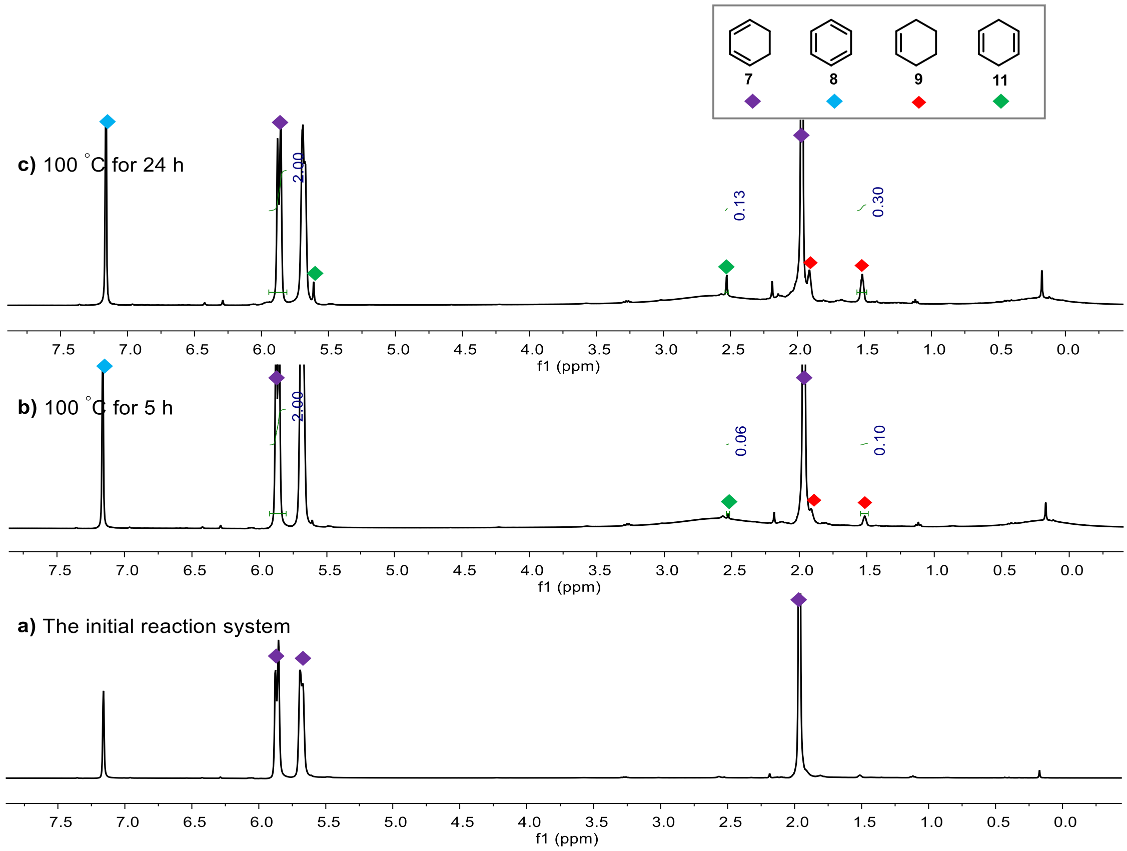

Figure S15. Disproportionation of **7** catalyzed by complex **3** (ppm: δ 0.0 ~ δ 7.5)

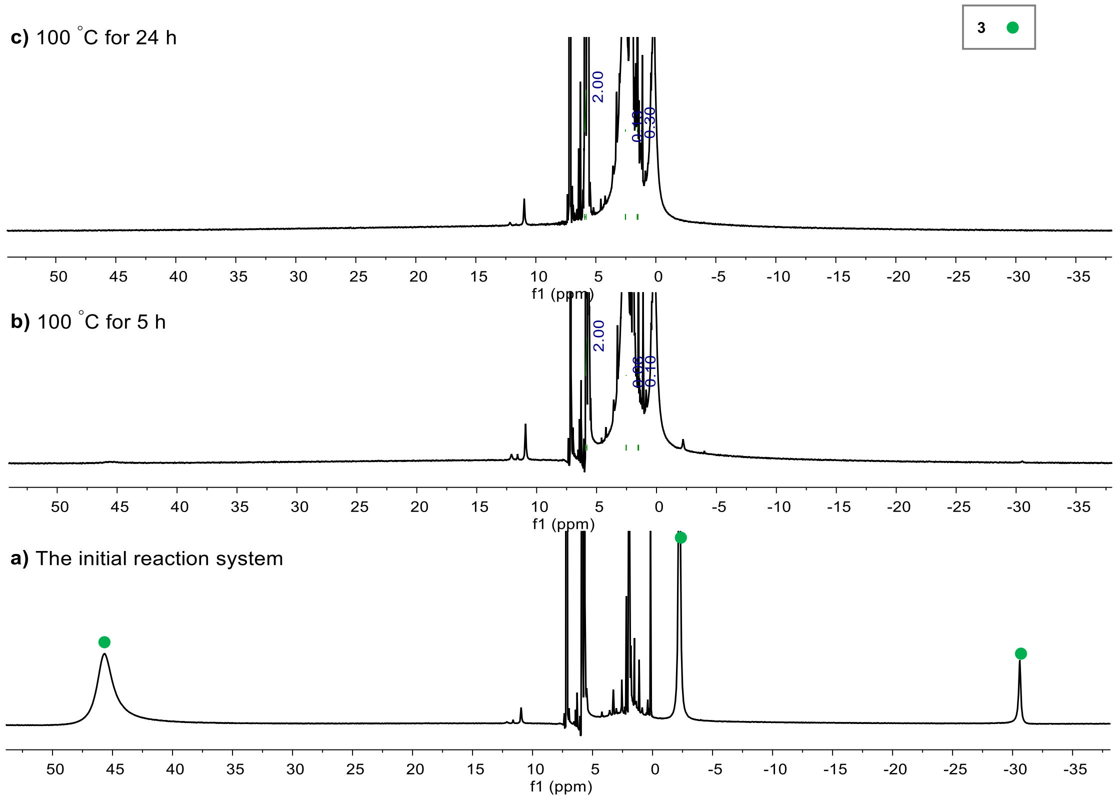

Figure S16. Disproportionation of **7** catalyzed by complex **3** (ppm: δ -35.0 ~ δ 50.0)

(3) Complex **4** as catalyst

In a N_2 glovebox, to a J. Young valve NMR tube was added complex **4** (21.3 mg, 0.03 mmol), C_6D_6 (0.5 mL) and **7** (24.0 mg, 0.30 mmol). The tube was heated at 100 °C for 24 h. The ^1H NMR analysis revealed that the conversion of **7** was 13% (**Figure S17**, **b**).

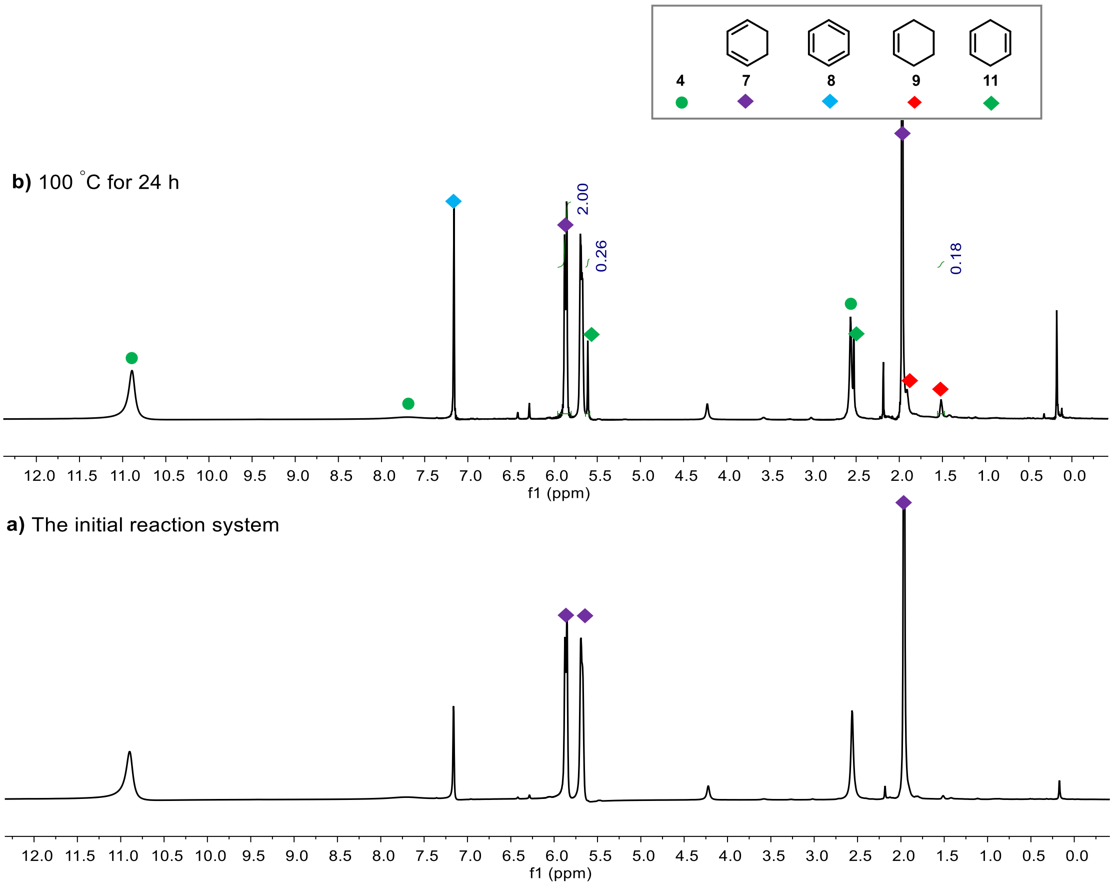

Figure S17. Disproportionation of **7** catalyzed by complex **4**

(4) $Mg[N(SiMe_3)Ar]_2$ as catalyst

In a N_2 glovebox, to a J. Young valve NMR tube was added $Mg[N(SiMe_3)Ar]_2$ (12.3 mg, 0.03 mmol), C_6D_6 (0.5 mL) and **7** (24.0 mg, 0.30 mmol). The tube was heated at 100 °C for 36 h. The ^{1}H NMR analysis revealed that disproportionation of **7** was not detected (**Figure S18**, **c**).

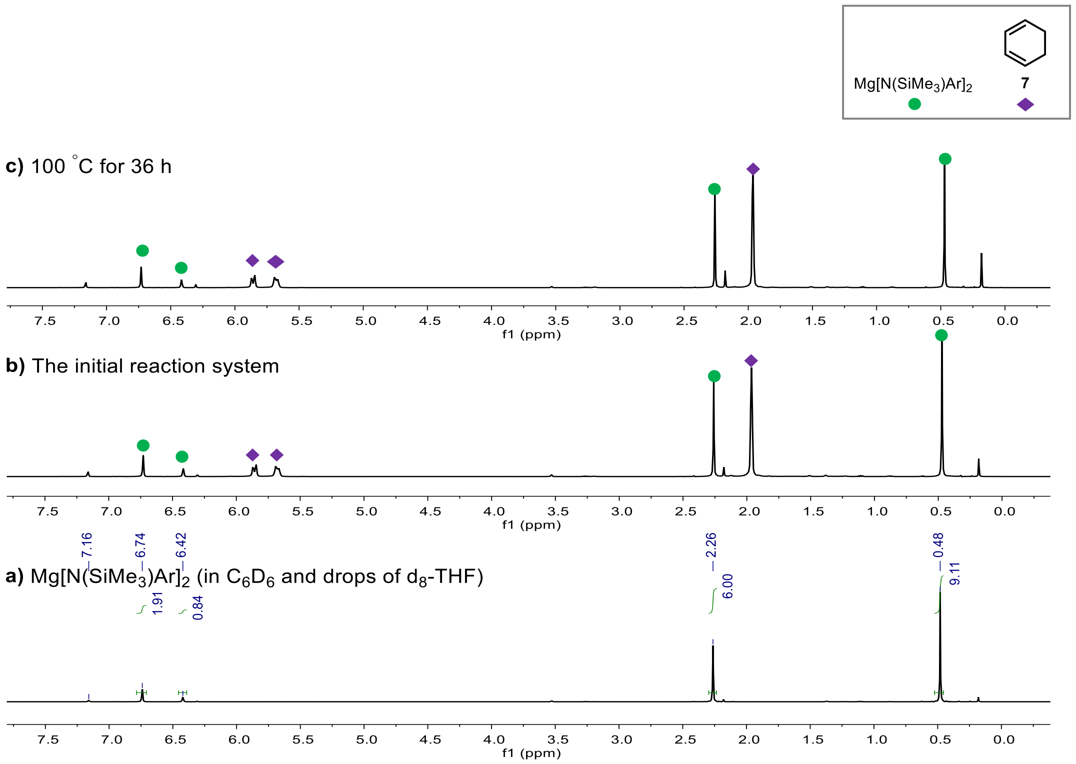

Figure S18. Disproportionation of **7** catalyzed by $Mg[N(SiMe_3)Ar]_2$

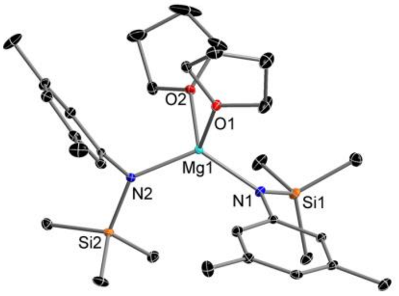

Figure S19. Crystal structure of $Mg(THF)_2[N(SiMe_3)Ar]_2$ with 10% thermal ellipsoids

(5) Li[N(SiMe$_3$)Ar] as catalyst

In a N_2 glovebox, to a J. Young valve NMR tube was added Li[N(SiMe$_3$)Ar] (6.0 mg, 0.03 mmol), C_6D_6 (0.5 mL) and **7** (24.0 mg, 0.30 mmol). The tube was heated at 100 °C for 19 h. The ^{1}H NMR analysis revealed that disproportionation of **7** was not detected (**Figure S20**, **b**).

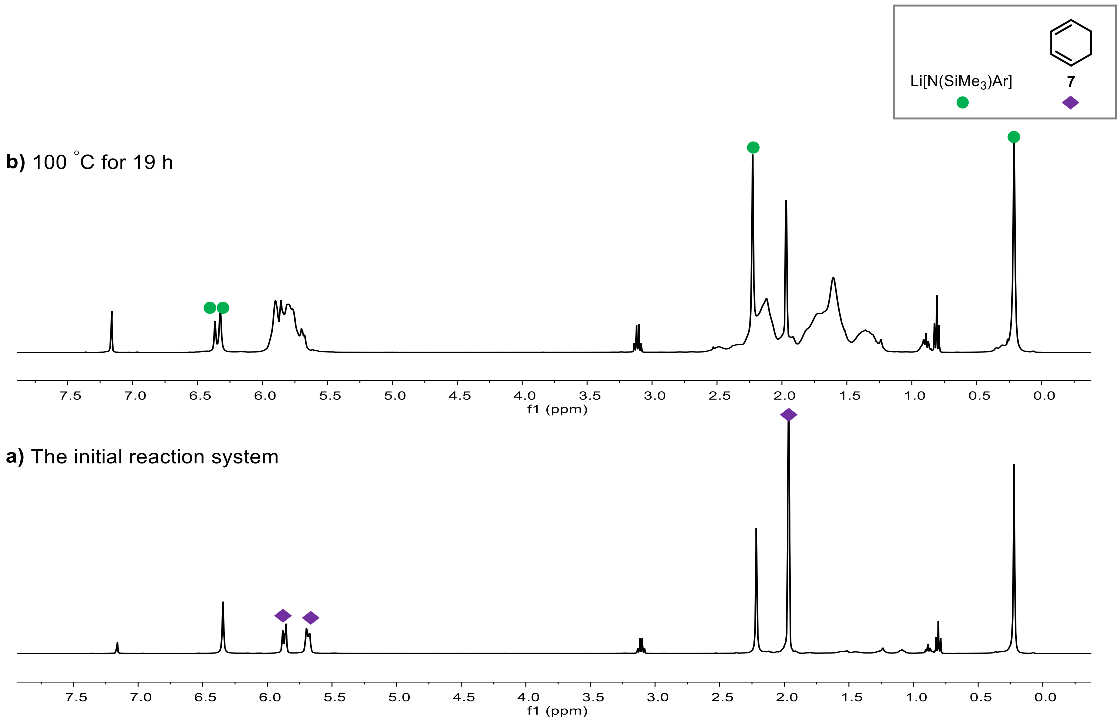

Figure S20. Disproportionation of **7** catalyzed by Li[N(SiMe$_3$)Ar]

4. Isomerization of Terminal Alkenes Catalyzed by Complex 5

4.1 Catalytic isomerization of allylbenzene (12)

In a N_2 glovebox, to a J. Young valve NMR tube was added complex **5** (31.8 mg, 0.03 mmol), C_6D_6 (0.5 mL) and allylbenzene (**12**) (35.4 mg, 0.30 mmol). The tube was heated at 60 °C for 12 h. The ^1H NMR analysis revealed that **12** was converted completely into the 1-phenyl-1-propene (**13**) (**Figure S21**, **b**). The product **13** was identified by comparing corresponding ^1H NMR spectrum with a commercial sample.

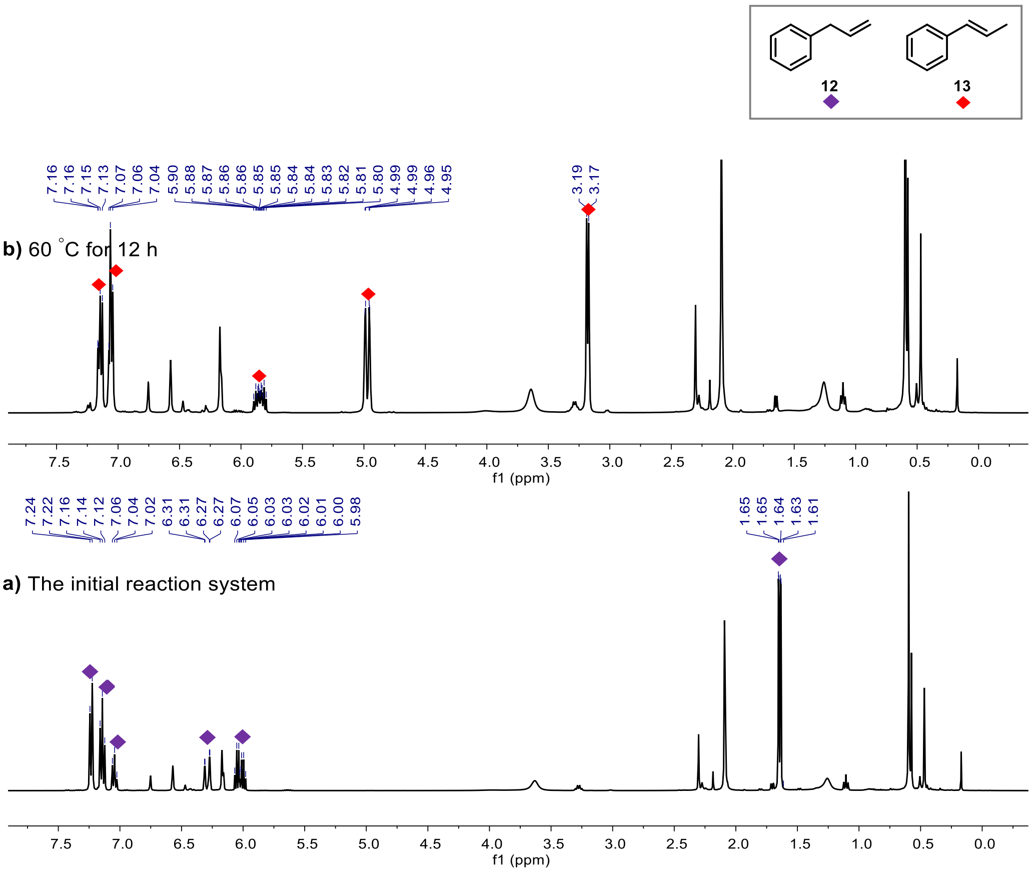

Figure S21. Catalytic isomerization of **12** catalyzed by complex **5**

4.2 Catalytic isomerization of 1-hexene (14)

In a N_2 glovebox, to a J. Young valve NMR tube was added complex **5** (31.8 mg, 0.03 mmol), C_6D_6 (0.5 mL) and 1-hexene (**14**) (35.4 mg, 0.30 mmol). The tube was heated at 100 °C for 12 h. The ^1H NMR analysis revealed that **14** was converted completely into the mixture of 2-hexene and 3-hexene (**15**) (**Figure S22**, **b**, conv. 97%). The products **15** were identified by comparing corresponding ^{13}C NMR spectrums described in the literatures (**Figure S23**) [5-6].

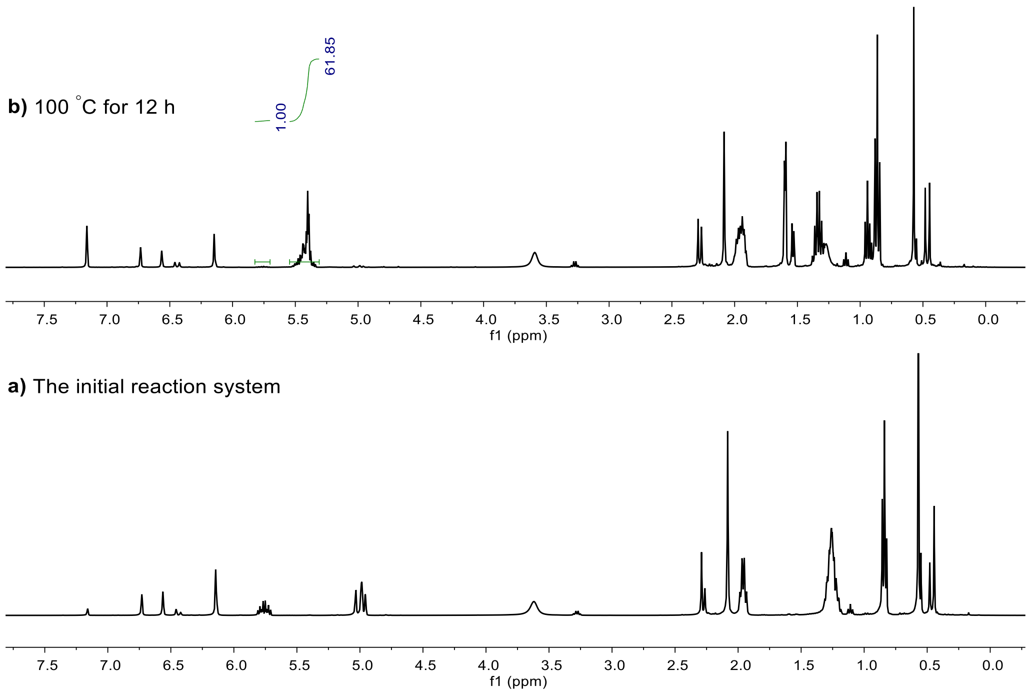

Figure S22. Catalytic isomerization of **14** catalyzed by complex **5**

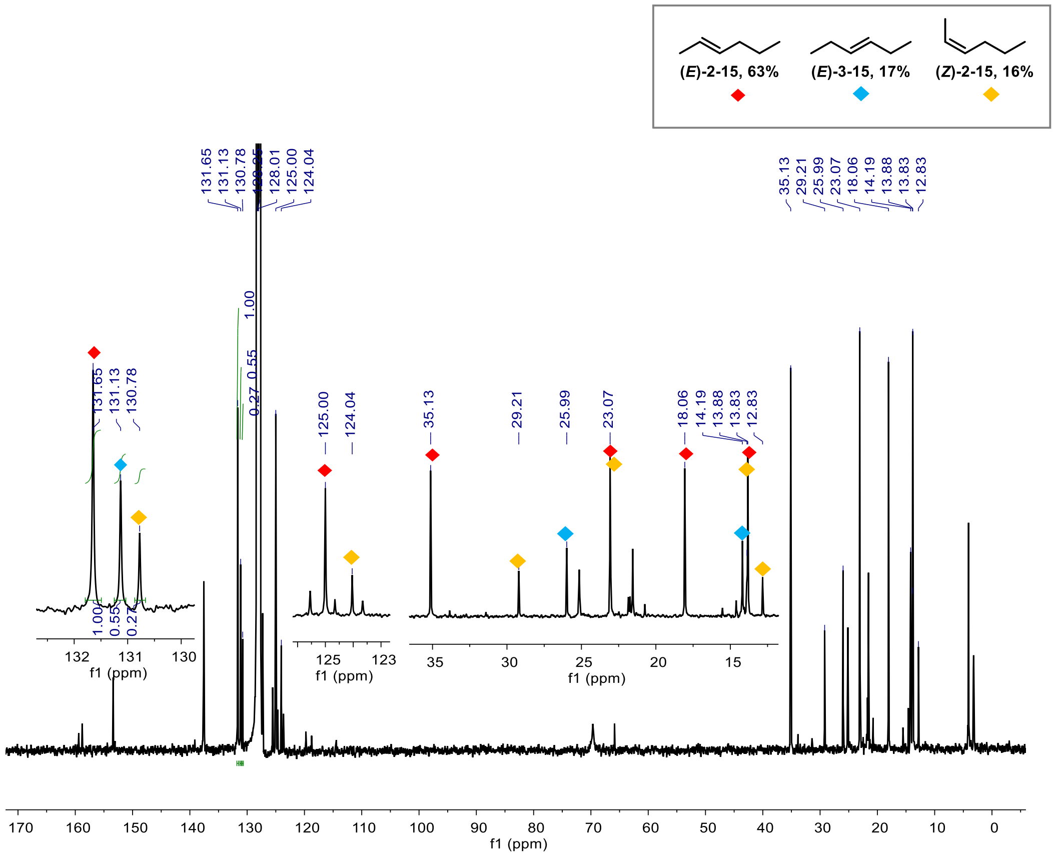

Figure S23. Inverse gated decoupled spectrum of rection mixture

5. Mechanistic Studies

5.1 Kinetic analysis for the catalytic disproportionation

The dependence of the initial rate on the catalyst (complex 5): In a N_2 glovebox, to a J. Young valve NMR tube was added complex **5** (as shown in **Table S1**), C_6D_6 (0.5 mL), and **7** (16.0 mg, 0.20 mmol). The tube was heated at 100 °C and monitored by NMR. Yields were obtained from the relative ^{1}H NMR integrations of THF (from catalyst, δ 3.75~3.50, O$CH_2$$CH_2$) and **9** (δ 1.57~1.47, $CHCH_2$$CH_2$). Figure **S25** showed that the initial rate of disproportionation depend on the concentration of catalyst **5** was the first order.

Table S1: Molarities of catalyst (complex **5**) and initial rates for each kinetic experiment

Entry	Catalyst (complex **5**) (mmol L^{-1})	**7** (mmol L^{-1})	Initial Rate (mmol L^{-1} min^{-1})
1	**16.0**	400.0	0.0156
2	**24.0**	400.0	0.0285
3	**32.0**	400.0	0.0360
4	**40.0**	400.0	0.0480

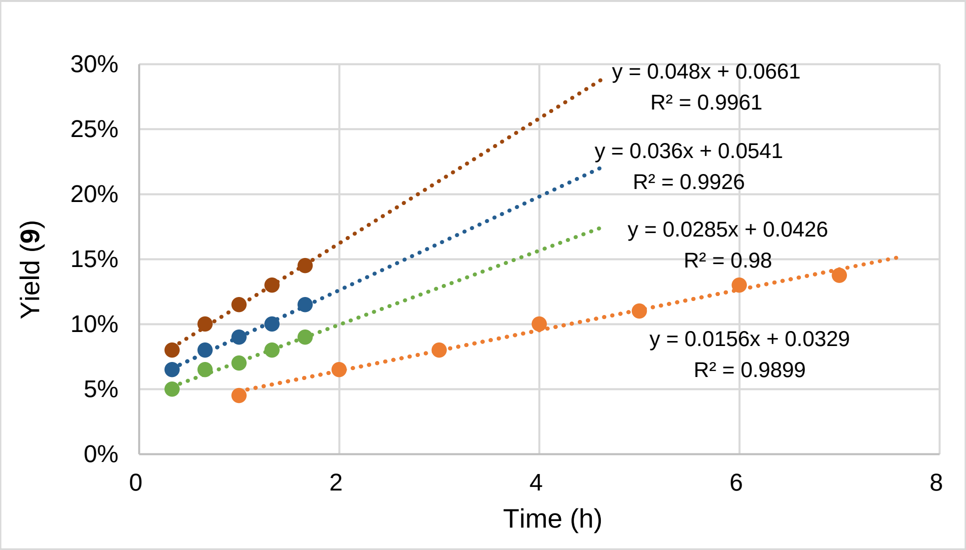

Figure S24. Time dependence on catalyst concentration for the catalytic disproportionation.

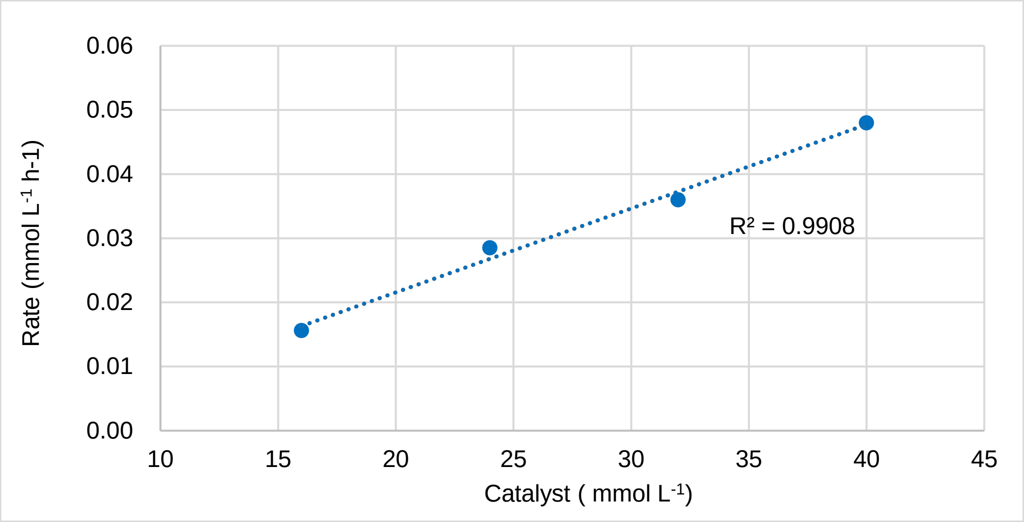

Figure S25. Initial reaction rate dependence on catalyst concentration for the catalytic disproportionation.

5.2 Intramolecular kinetic isotope effect experiment for the catalytic isomerization

In a N_2 glovebox, to a J. Young valve NMR tube was added complex **5** (21.2 mg, 0.02 mmol), C_6D_6 (0.5 mL) and **16-*d₁*** (39.0 mg, 0.20 mmol). The tube was allowed to proceed at room temperature for 2 h, after then treated with silica gel column chromatography (hexane as the eluent) to afford the mixture of unreacted starting material **16-*d₁*** and product **17-*d₁***. The KIE value was determined as 2.07 by ^{1}H NMR analysis (Conv. of **16-*d₁***: 14%, obtained from the relative ^{1}H NMR integrations of substrate and product).

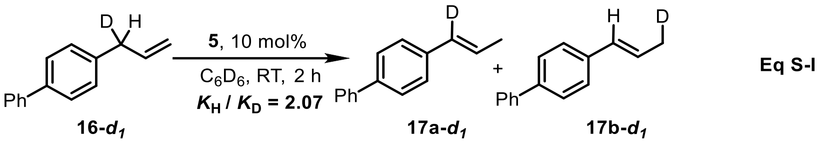

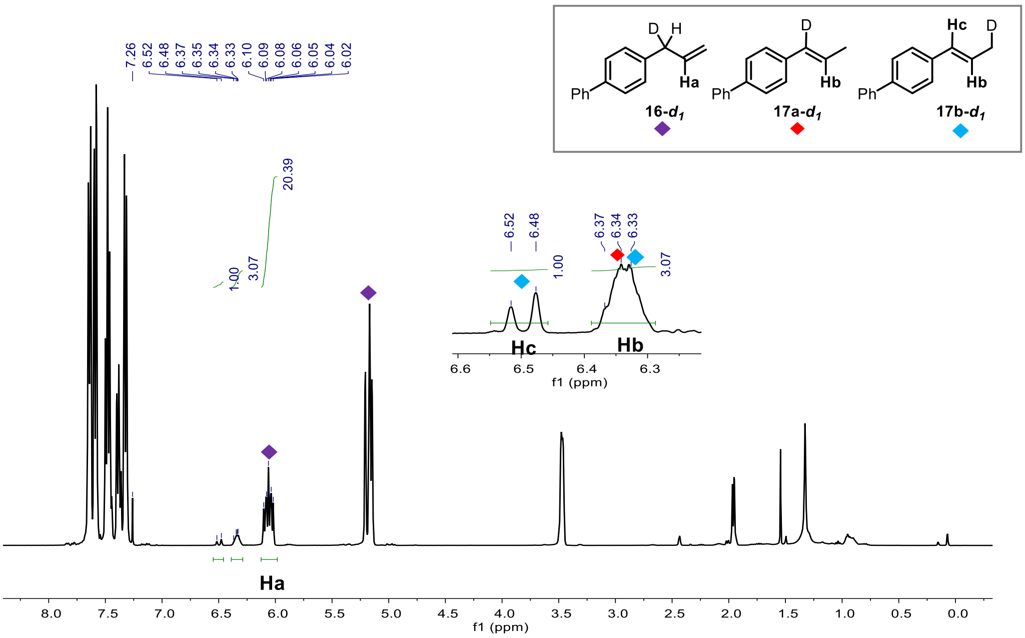

Figure S26. ^{1}H NMR spectrum of the mixture of **16-*d₁*** and **17-*d₁*** in $CDCl_3$

5.3 Intermolecular kinetic isotope effect experiment for the catalytic isomerization

In a N_2 glovebox, to a J. Young valve NMR tube was added complex **5** (21.2 mg, 0.02 mmol), C_6D_6 (0.5 mL), and **16** (38.8 mg, 0.20 mmol). The tube was allowed to proceed at room temperature and monitored by ^{1}H NMR every 15 min. The reaction of **16-*d*$_2$** was carried out under exact same conditions. The kinetic isotope effect was proved to be 1.39 (**Figure S27**). The yields of **17** and **17-*d*$_2$** were obtained from the relative ^{1}H NMR integrations of substrate and product.

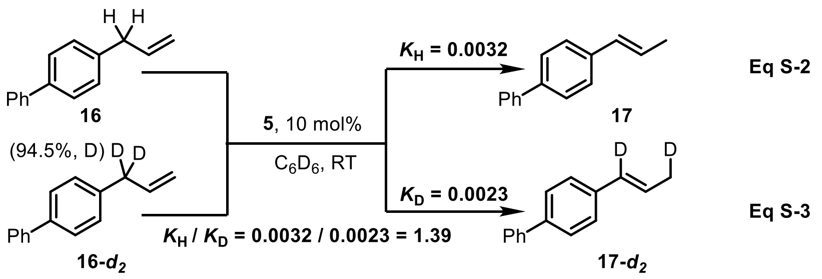

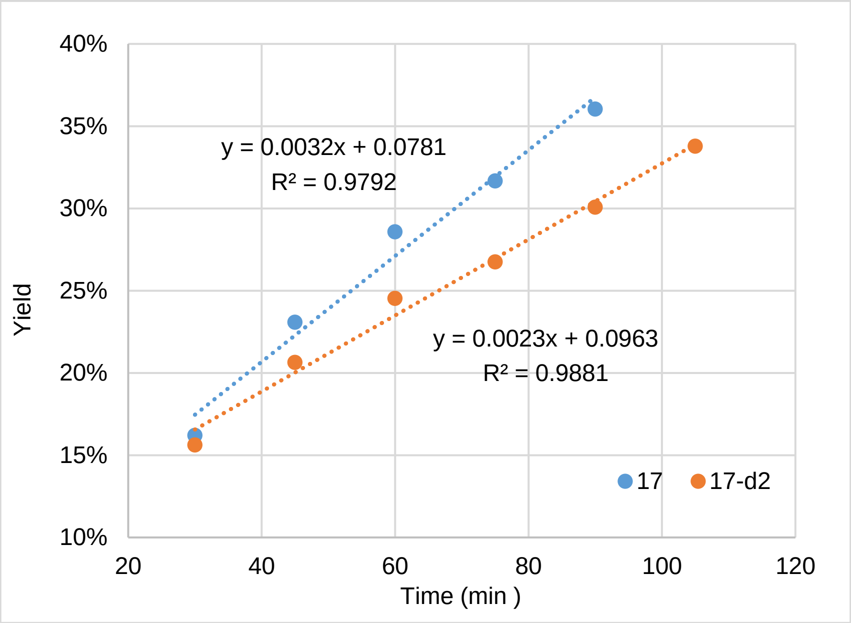

Figure S27. Initial rates of catalytic isomerization of **16** and **16-*d*$_2$**

The products **17** and **17-*d*$_2$** (**Figure S28-Figure S31**) were isolated after the reaction mixture (**Eq S-2** and **Eq S-3**) proceeding at room temperature for 24 h, then purification directly by silica gel column chromatography (Hexane as the eluent).

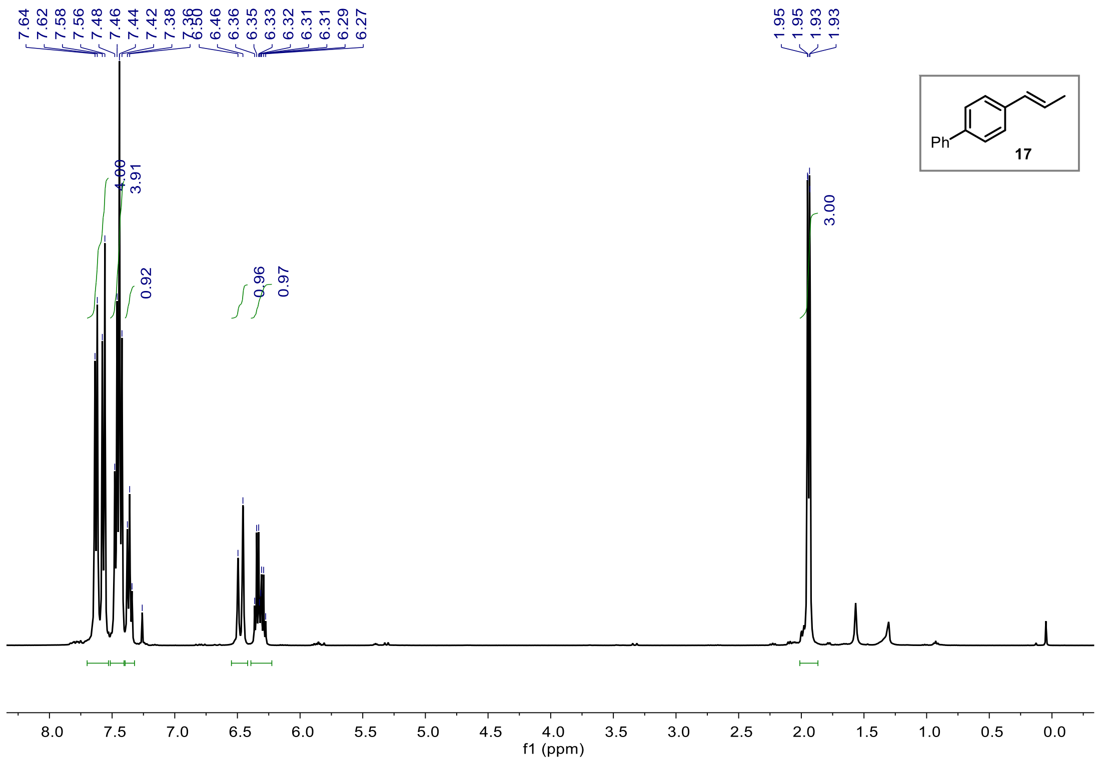

Figure S28. The ^{1}H NMR spectrum of **17** in **Eq-S2** ($CDCl_3$)

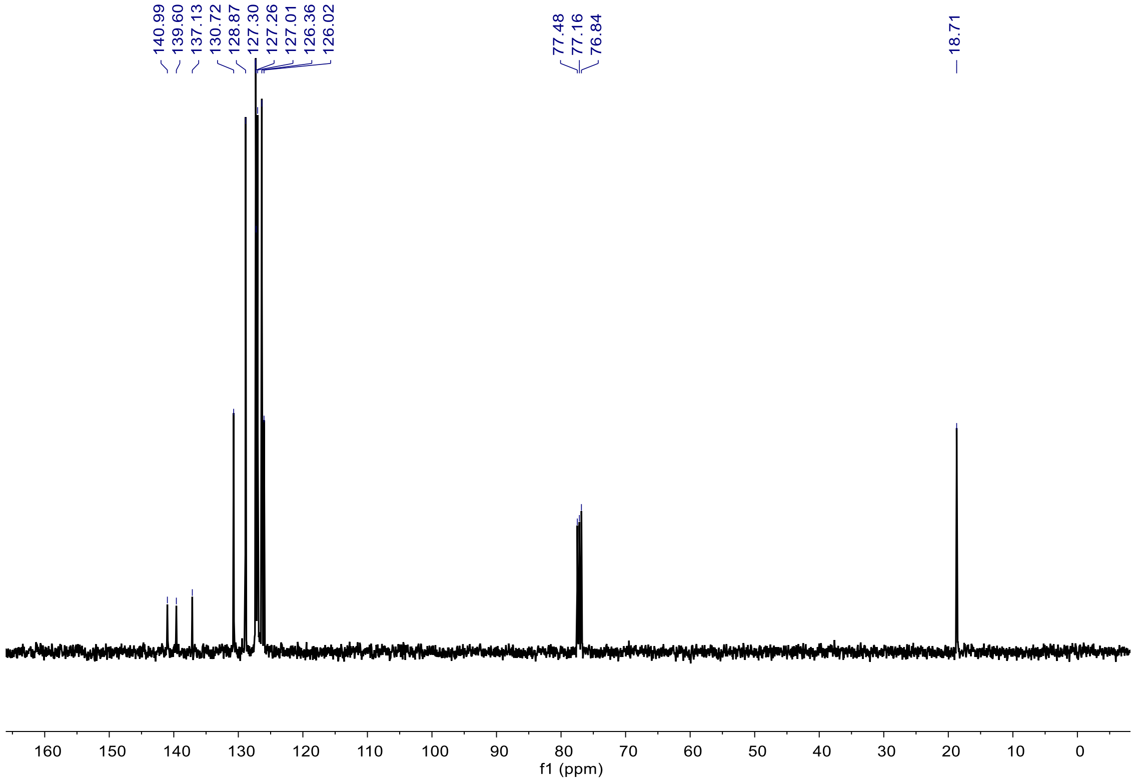

Figure S29. The ^{13}C NMR spectrum of **17** in **Eq-S2** ($CDCl_3$)

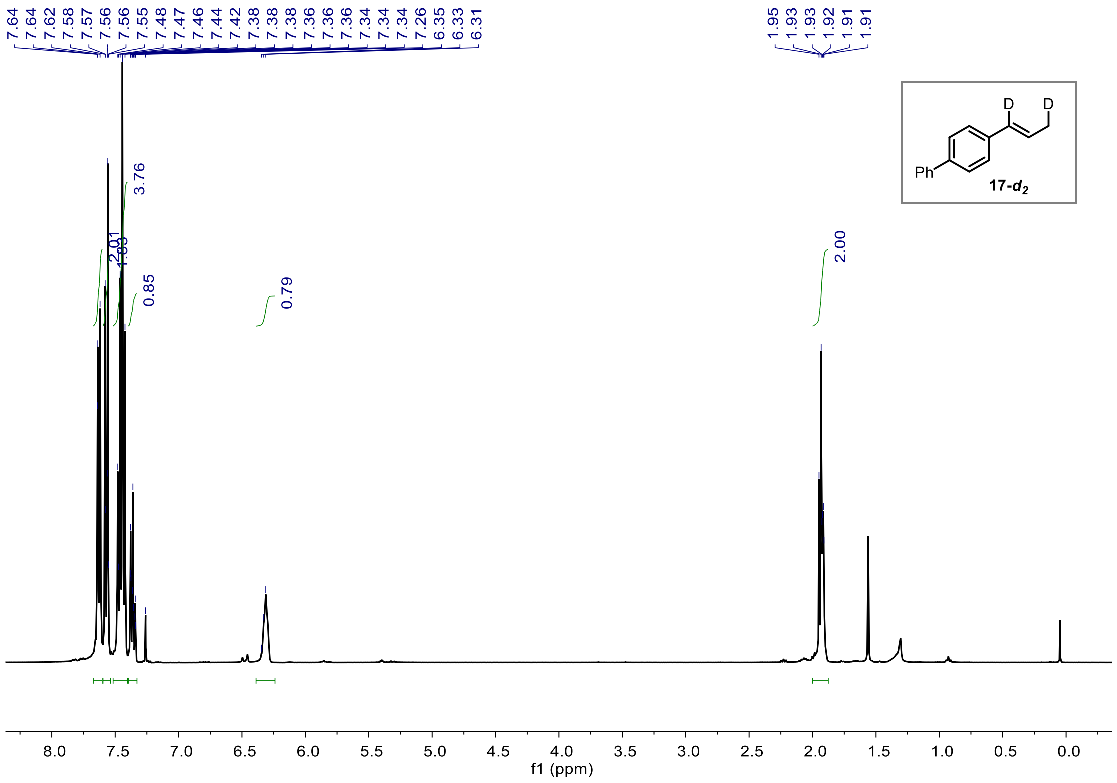

Figure S30. The ^{1}H NMR spectrum of ***17-d₂*** in **Eq-S3** ($CDCl_3$)

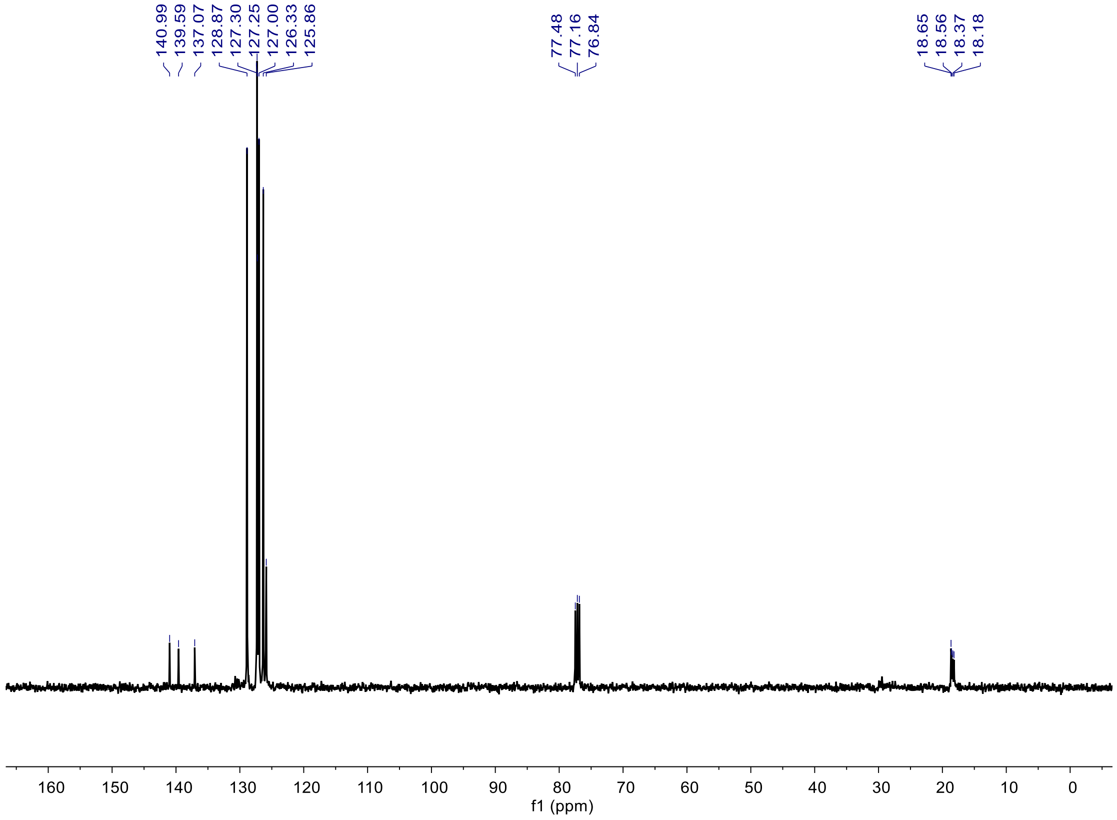

Figure S31. The ^{13}C NMR spectrum of ***17-d₂*** in **Eq-S3** ($CDCl_3$)

6. NMR Data

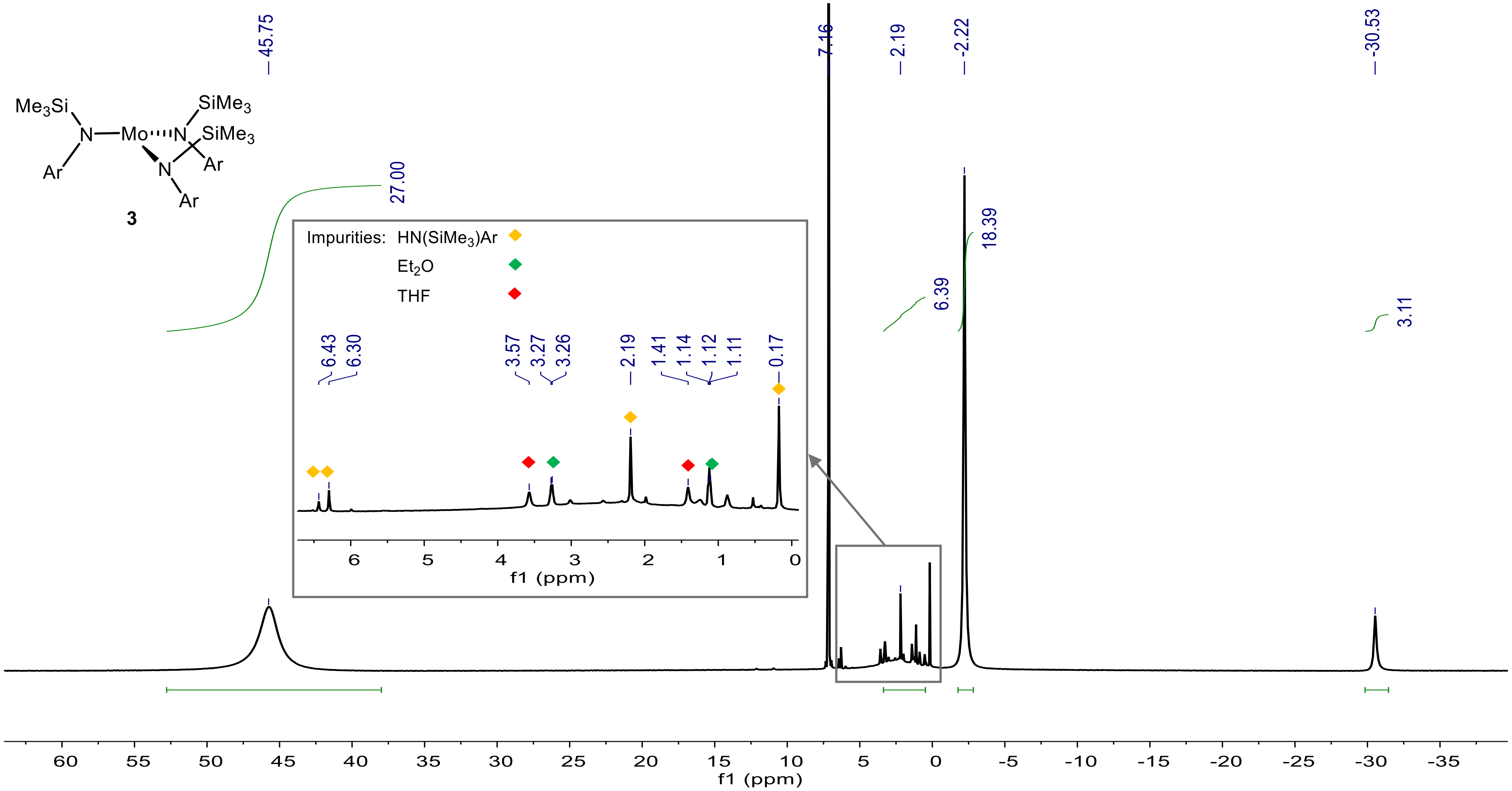

Figure S32. The ^{1}H NMR spectrum of complex **3** (C_6D_6)

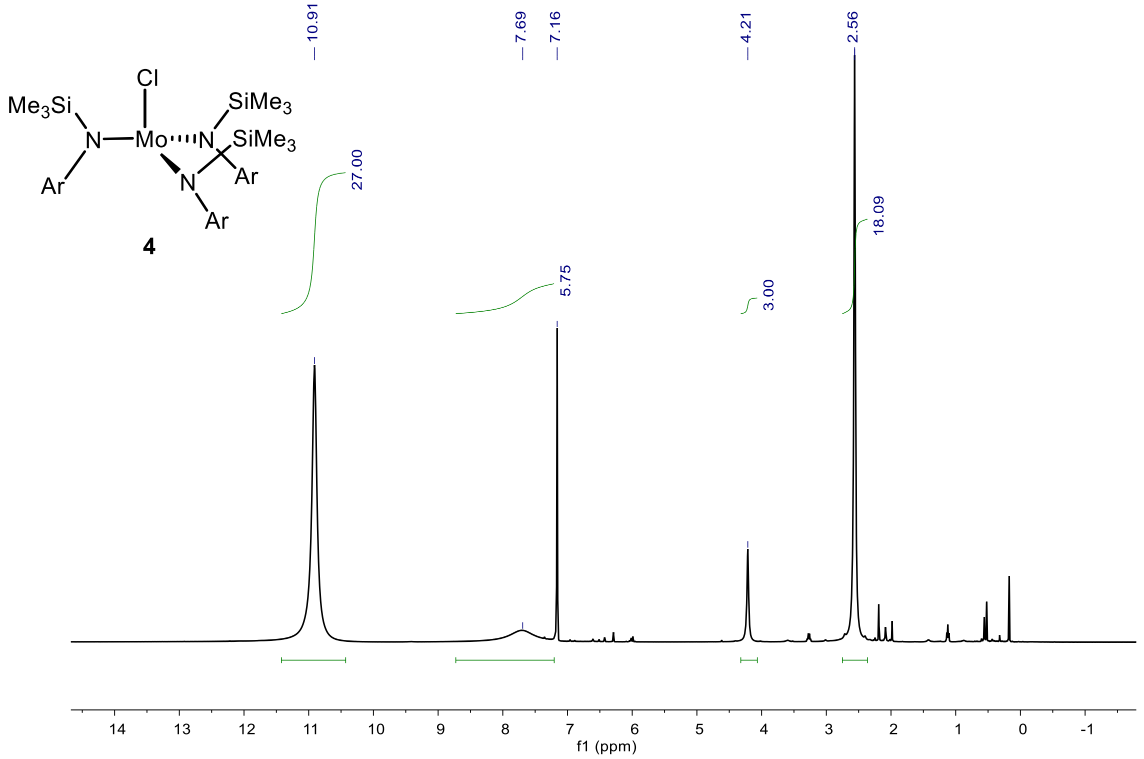

Figure S33. The ^{1}H NMR spectrum of complex **4** (C_6D_6)

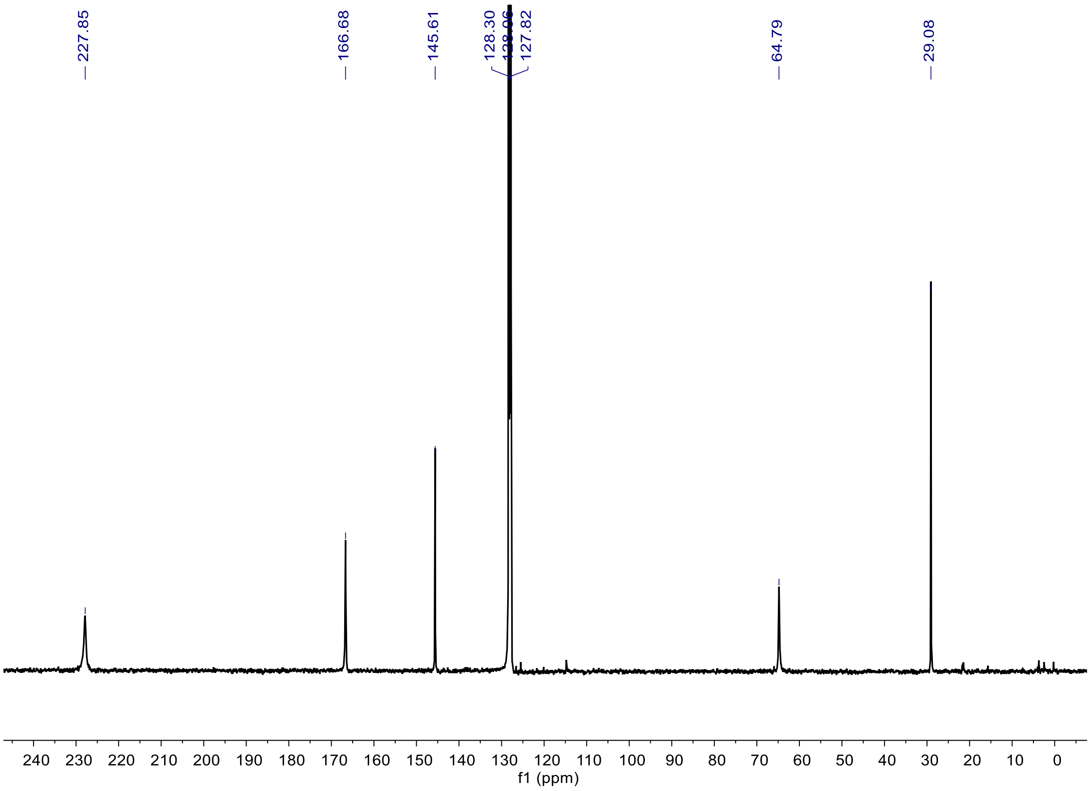

Figure S34. The ^{13}C NMR spectrum of complex **4** (C_6D_6)

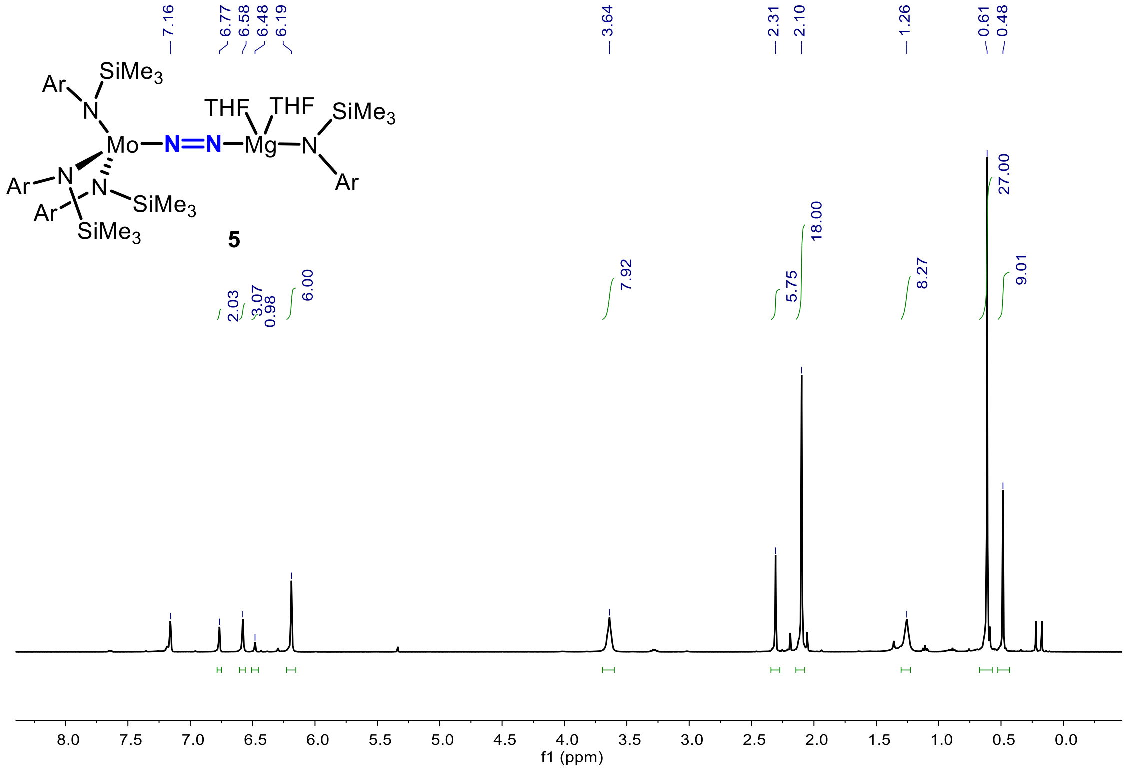

Figure S35. The ^{1}H NMR spectrum of complex **5** (C_6D_6)

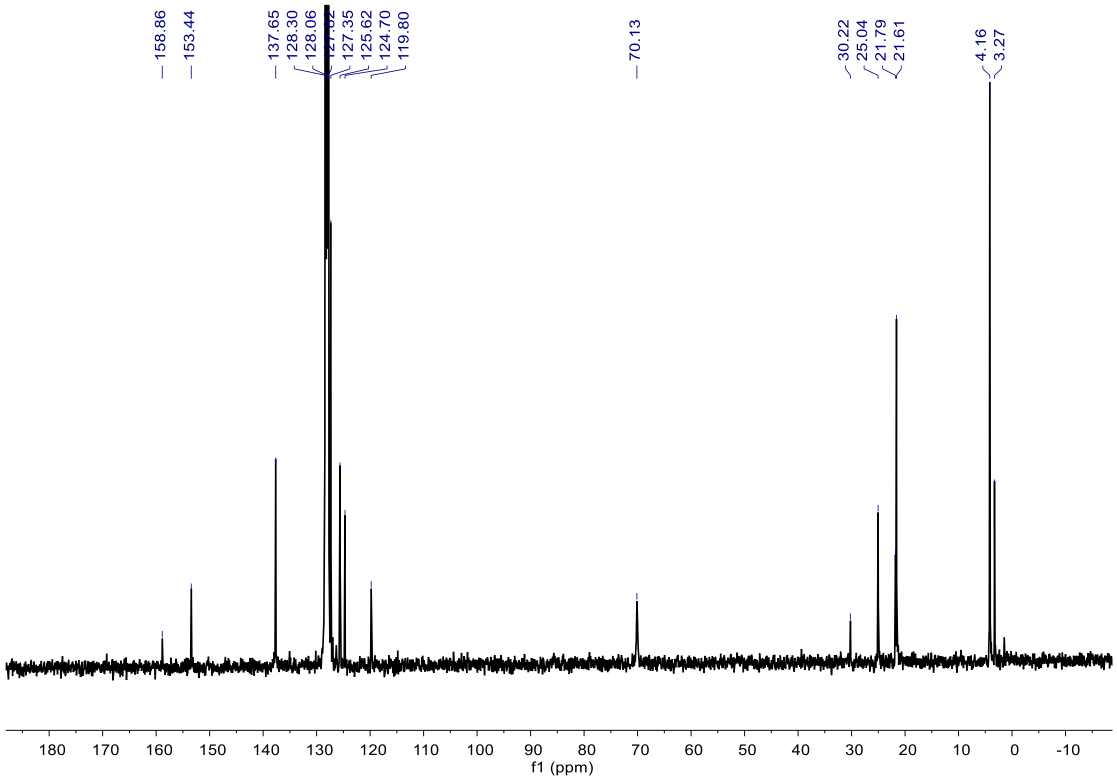

Figure S36. The ^{13}C NMR spectrum of complex **5** (C_6D_6)

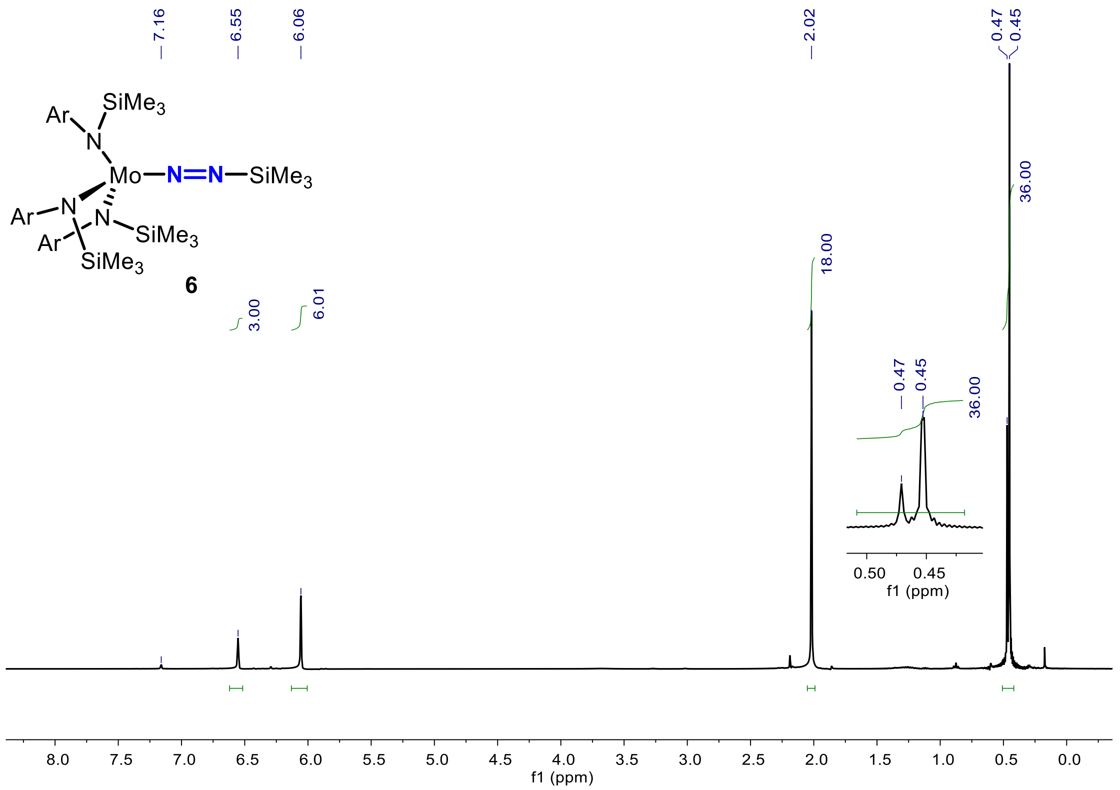

Figure S37. The ^{1}H NMR spectrum of complex **6** (C_6D_6)

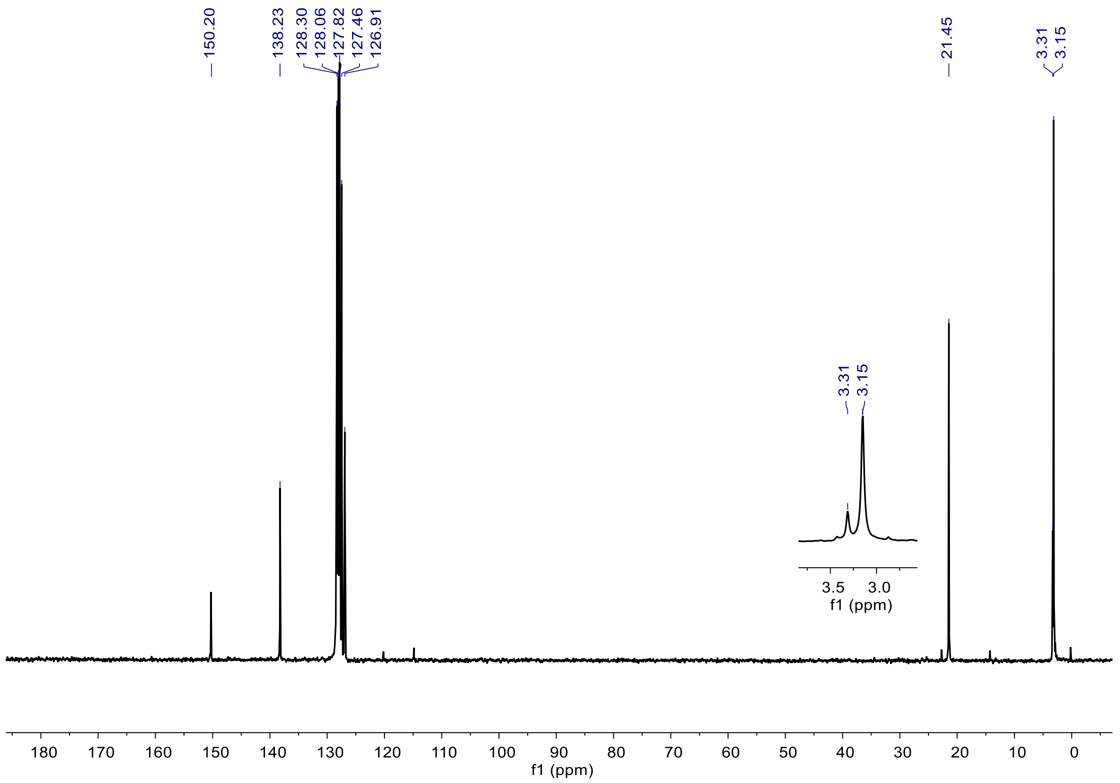

Figure S38. The ^{13}C NMR spectrum of complex **6** (C_6D_6)

7. References

1. Zhang W, Lu YY and Moore JS. Preparation of a trisamidomolybdenum(VI) propylidyne complex—A highly active catalyst precursor for alkyne metathesis. *Org Synth* 2007; **84**: 163-176.

2. Nagae H, Hato W and Kawakita K *et al*. Arylimido-Bridged Dinuclear Ti(μ-NAr)$_2$Ti Scaffold for Alkyne Insertion into the *ortho*-C-H Bond of Arylimido Ligands. *Chem Eur J* 2017; **23**: 586-596.

3. Yin GY, Wu YC and Liu GS. Scope and Mechanism of Allylic C-H Amination of Terminal Alkenes by the Palladium/PhI(OPiv)$_2$ Catalyst System: Insights into the Effect of Naphthoquinone. *J Am Chem Soc* 2010; **132**: 11978-11987.

4. Murai M, Nishimura K and Takai K. Palladium-catalyzed double-bond migration of unsaturated hydrocarbons accelerated by tantalum chloride. *Chem Commun* 2019; **55**: 2769-2772.

5. Schwieger S, Herzog R and Wagner C *et al*. Platina-β-diketones as catalysts for hydrosilylation and their reactivity towards hydrosilanes. *J Organomet Chem* 2009; **694**: 3548-3558.

6. Wang YL, Qin C and Jia XQ *et al*. An Agostic Iridium Pincer Complex as a Highly Efficient and Selective Catalyst for Monoisomerization of 1-Alkenes to trans-2-Alkenes. *Angew Chem Int Ed* 2017; **56**: 1614-1618.
